# Structural Premise of Selective Deubiquitinase USP30 Inhibition by Small-Molecule Benzosulfonamides

**DOI:** 10.1016/j.mcpro.2023.100609

**Published:** 2023-06-28

**Authors:** Darragh P. O'Brien, Hannah B.L. Jones, Franziska Guenther, Emma J. Murphy, Katherine S. England, Iolanda Vendrell, Malcolm Anderson, Paul E. Brennan, John B. Davis, Adán Pinto-Fernández, Andrew P. Turnbull, Benedikt M. Kessler

**Affiliations:** 1 Target Discovery Institute, Centre for Medicines Discovery, Nuffield Department of Medicine, University of Oxford, Oxford, Oxfordshire, UK; 2 ARUK-Oxford Drug Discovery Institute, Centre for Medicines Discovery, Nuffield Department of Medicine, University of Oxford, Oxford, Oxfordshire, UK; 3 Waters Corporation, Wilmslow, Cheshire, UK; 4 Chinese Academy of Medical Sciences Oxford Institute, Nuffield Department of Medicine, University of Oxford, Oxford, Oxfordshire, UK; 5 Cancer Research Horizons, Francis Crick Institute, London, UK

**Keywords:** ubiquitin-specific protease 30, small-molecule benzosulfonamides, activity-based protein profiling, hydrogen–deuterium exchange mass spectrometry, deubiquitinylases, Parkinson's disease, mitophagy, drug discovery, structure-activity relationships, molecular docking simulations

## Abstract

Dampening functional levels of the mitochondrial deubiquitylating enzyme Ubiquitin-specific protease 30 (USP30) has been suggested as an effective therapeutic strategy against neurodegenerative disorders such as Parkinson’s Disease. USP30 inhibition may counteract the deleterious effects of impaired turnover of damaged mitochondria, which is inherent to both familial and sporadic forms of the disease. Small-molecule inhibitors targeting USP30 are currently in development, but little is known about their precise nature of binding to the protein. We have integrated biochemical and structural approaches to gain novel mechanistic insights into USP30 inhibition by a small-molecule benzosulfonamide-containing compound, USP30_inh_. Activity-based protein profiling mass spectrometry confirmed target engagement, high selectivity, and potency of USP30_inh_ for USP30 against 49 other deubiquitylating enzymes in a neuroblastoma cell line. *In vitro* characterization of USP30_inh_ enzyme kinetics inferred slow and tight binding behavior, which is comparable with features of covalent modification of USP30. Finally, we blended hydrogen–deuterium exchange mass spectrometry and computational docking to elucidate the molecular architecture and geometry of USP30 complex formation with USP30_inh_, identifying structural rearrangements at the cleft of the USP30 thumb and palm subdomains. These studies suggest that USP30_inh_ binds to this thumb–palm cleft, which guides the ubiquitin C terminus into the active site, thereby preventing ubiquitin binding and isopeptide bond cleavage, and confirming its importance in the inhibitory process. Our data will pave the way for the design and development of next-generation inhibitors targeting USP30 and associated deubiquitinylases.

## Highlights

• The small-molecule benzosulfonamide USP30_inh_ is highly potent and selective for neuronal USP30.
• The inhibitor binds USP30 in a slow and tight manner and displays kinetic properties consistent with covalent attachment to USP30, despite its noncovalent design.
• We identified regions within USP30 that undergo dramatic structural and conformational rearrangements by USP30_inh_, preventing Ub binding and decrease DUB activity.
• Insights into USP30 inhibitory mechanistics will guide the development of next-generation inhibitors, which has relevance in neurodegeneration.

## Graphical Abstract

**Figure:**
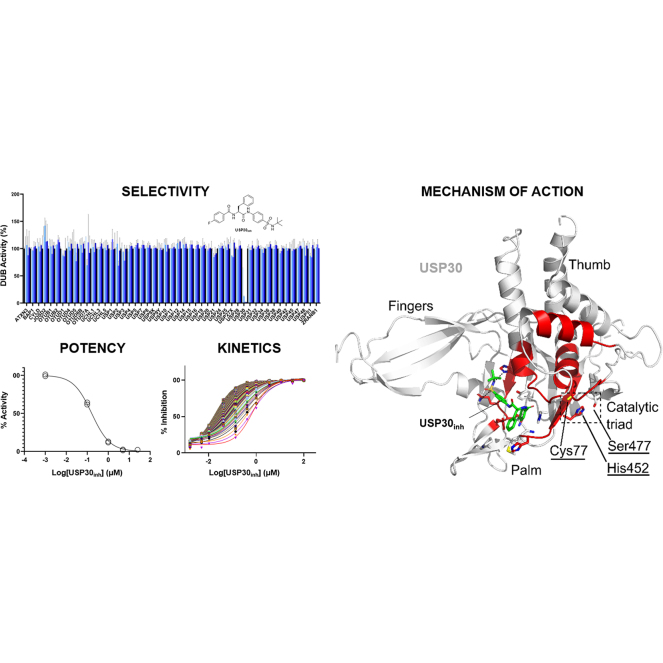

## In Brief

We describe the dynamic structural interplay between USP30 and the small-molecule benzosulfonamide-containing compound, USP30_inh_. The inhibitor is highly potent and selective against endogenous USP30, binding in a slow and tight manner. Upon complex formation, significant perturbations occur in the conformational dynamics of the USP30 thumb–palm cleft region, proximal to its catalytic cysteine. Inhibitor binding here would sterically clash with Ub, preventing its binding and isopeptide bond cleavage. Our results help drive the design of the next generation of USP30 inhibitors.

Ubiquitination is essential to protein quality control, homeostasis, and life span (1). Damaged proteins are flagged for removal from cells with the covalent addition of ubiquitin (Ub), a small highly conserved 76-amino acid protein that is widely expressed across eukaryotic cell types (2, 3). This molecular “kiss of death” proceeds through the coordinated action of E1, E2, and E3 ligase enzymes, with targeted protein substrates conjugated by way of an isopeptide bond to either a single Ub molecule (monoubiquitination) or several repeating units (polyubiquitination) (4). Such modifications participate and control a vast array of cellular processes through proteolytic or nonproteolytic means, as seen in proteostasis, endocytic trafficking, NF-κB activation and inflammation, translation, DNA repair, and control of cell cycle processes (5). Besides the Ub–proteasome system subfamily, proteins selected for degradation can also be trafficked through the autophagy–lysosome pathway. In damaged mitochondria, such autophagic clearance of impaired proteins occurs through a highly selective and dedicated mechanism termed “mitophagy.” Several key players work tirelessly in the dysregulated mitochondrion to maintain overall cell integrity and survival, including the mitochondrial outer membrane (MOM)–associated Ub serine/threonine kinase PINK1 and the cytoplasmic E3 ligase Parkin (6). PINK1 phosphorylates Ub species on damaged proteins accumulating on the MOM, flagging them for elimination. This in turn recruits and activates endogenous Parkin (also by phosphorylation), initiating a hyperubiquitination cascade that gives the green light for mitophagy to proceed in a specialized autophagosome structure (7). Deubiquitinating enzymes (DUBs) counteract the actions of E3 ligases by removing Ub modifications, and several of these Ub-specific proteases (USPs) have been shown to oppose Parkin activity (8, 9). Of these, USP30 is the predominant active DUB to be directly implicated in mitophagy to-date, primarily due to its localization on the MOM, whilst also being linked to pexophagy and oxygen metabolism because of its widespread expression on peroxisomes (10). Interestingly, both Parkin and USP30 share an unusual preference for Lys6-linked Ub chains, the mitophagic importance of which has yet to be conclusively deciphered (11, 12).

Impaired mitophagy and oxidative stress have adverse roles in neurodegeneration, with both being linked to familial and sporadic forms of Parkinson’s Disease (PD) (13, 14); PINK1–Parkin-mediated mitophagy limits the build-up of toxic mitochondrial species in PD, and loss-of-function mutations occurring in the PINK1 and PRKN genes result in the progressive depletion of dopaminergic neurons of the basal ganglia and a rare hereditary form of juvenile Parkinsonism (15, 16). As USP30 antagonizes mitophagy through ubiquitination, its inhibition has been proposed as a novel therapeutic strategy to enhance mitochondrial turnover and clear damaged mitochondrial proteins, providing a much-needed strategy to improve outcomes in PD and other neurodegenerative disorders. Several small-molecule inhibitors targeting USP30 are in the pipeline, including phenylalanine derivatives, *N*-cyano pyrrolidines, and natural products (17, 18, 19). Perhaps those with the greatest potential, however, are a family of benzosulfonamides, most notably, the family member of CAS number 2242582-40-5 and hereon in referred to as “USP30_inh_”, which has been shown to boost mitophagy in dopaminergic neurons of PD patients by downregulating USP30 (20, 21). Little is currently known, however, regarding the intricacies of its noncovalent attachment to USP30 itself, and the structural basis of its inhibitory action. X-ray crystallography has recently provided structures of both human and zebrafish USP30, but these are solely in the context of attachment to Lys6-linked di-Ub moieties (11, 12). Hydrogen–deuterium exchange mass spectrometry (HDX-MS) is a complementary biophysical tool that can provide unique insights into protein structure, stability, dynamics, and function (22). In direct contrast to the static snapshot provided by the crystal structure, HDX-MS monitors the conformational dynamics of a system *in solution*, enabling the analysis of “proteins in motion.” The technique has shown particular utility for the rapid and reliable identification of small-molecule binding pockets on proteins (23). Whilst lacking the resolution of a crystal structure, the highly informative data obtained from HDX-MS can be combined with orthogonal structural, computational, biochemical, and/or biophysical techniques to define structure–activity relationships (SARs) and to direct drug discovery campaigns.

As such, we have integrated several biophysical and structural approaches to help clarify the molecular and structural interplay of USP30_inh_ binding to USP30. The endogenous cellular selectivity of USP30_inh_ for USP30 inhibition was confirmed using activity-based protein profiling mass spectrometry (ABPP-MS) against a panel of 49 other endogenous DUBs in neuronal SH-SY5Y cells. Biolayer interferometry showed that USP30_inh_ binds to USP30 in a slow and tight manner, which intriguingly, is consistent with the profile of covalent inhibition, despite it being a noncovalent binder. Finally, we combined HDX-MS and molecular docking simulations to elucidate the conformational dynamics and spatial preferences of USP30_inh_ binding, identifying key residues in the inhibitory process itself. USP30_inh_ induces conformational and structural rearrangements at the cleft of the USP30 thumb and palm subdomains, in a region encompassing its catalytic residues and the site of Ub binding. We postulate that these phenomena underlie the mechanism of inhibition of USP30_inh_. Dampening USP30 pharmacologically may represent a tractable treatment for PD and other mitophagy-related disorders. As no previous attempts to investigate the molecular basis of compound USP30_inh_ binding to USP30 have been reported, our data will be instrumental in the development of rational next-generation inhibitors against USP30 and related DUBs.

## Experimental Procedures

### Purity

All compounds were >95% pure by HPLC analysis (supplemental Fig. S1). USP30_inh_ refers to CAS number 2242582-40-5, which may be referred to as USP30 inhibitor compound **39** elsewhere.

### Experimental Design and Statistical Rationale

The inhibition of USP30_inh_ against endogenous USP30 was probed in SH-SY5Y cells using ABPP-MS. Cell extracts were treated with a range of inhibitor concentrations and quantified using label-free quantification and data-independent acquisition (DIA). All measurements were performed in biological duplicate. To decipher the conformational dynamic of inhibitor binding to a recombinant version of the protein, HDX-MS was performed. A USP30 sequence coverage map was generated following triplicate analysis of peptides acquired by MS^e^. Triplicate measurements were also obtained for both apo- and holo-USP30 across several deuterium-labeling time points. To estimate the statistical significance of deuterium uptake levels for apo- and holo-USP30, the average deuterium uptake value across triplicate measurements was first calculated for each USP30 peptide. We determined *p* values using a two-tailed one-sample Student’s *t* test calculated for all peptides across states, which allowed us to readily identify statistically significant changes in the deuterium uptake patterns of USP30 induced by inhibitor binding.

#### ABPP Assay

##### Hemagglutinin–Ub–Propargylamine Synthesis

Hemagglutinin (HA)–Ub–propargylamine (PA) synthesis was carried out as previously described (24, 25). Briefly, a pTYB construct was used to express Ub (Gly76del) in *Escherichia coli*. The Ub was tagged with a HA tag on the N terminus, and an intein–chitin binding domain on the C terminus. *E. coli* lysis was performed by sonication in 50 mM Hepes, 150 mM NaCl, 0.5 mM DTT. The protein was then bound to chitin bead slurry and incubated with 100 mM MesNa overnight (37 °C with agitation) to form HA–Ub–MesNa. The HA–Ub–MesNa was then incubated for 20 min with 250 mM PA (room temperature with agitation) and desalted to remove excess PA, resulting in the reactive activity-based probe HA–Ub–PA.

##### Cell Culture and Lysis

SH-SY5Y cells were cultured at 37 °C, 5% CO_2_ in Eagle’s minimum essential medium and Ham’s F12 nutrient mix (1:1), supplemented with 15% fetal bovine serum, 1% nonessential amino acids, and 2 mM Glutamax. Cells were collected by washing with PBS, followed by scraping in PBS and centrifugation at 200*g*. Cells were lysed in 50 mM Tris base, 5 mM MgCl_2_.6 H_2_O, 0.5 mM EDTA, 250 mM sucrose, 1 mM DTT by vortexing with acid washed beads (1:1 v/v) ten times (30 s vortexing, 1 min break on ice). Lysates were clarified at 600*g* for 10 min at 4 °C. Lysate protein concentration was then determined by bicinchoninic acid.

##### Inhibitor Selectivity With HA–Ub–PA Immunoprecipitation

HA–Ub–PA protein complexes were immunoprecipitated and analyzed using LC–MS/MS as previously described (26). USP30_inh_ or dimethyl sulfoxide (DMSO) was incubated with 500 μg of SH-SH5Y protein lysate for 1 h at 37 °C. HA–Ub–PA was then incubated with the USP30_inh_-treated lysates for 45 min at 37 °C at a protein ratio of 1:200 (w/w). The reaction was quenched with the addition of 0.4% SDS and 0.5% NP-40 (IGEPAL CA-630) and diluted to 0.5 mg/ml with 50 mM Tris, 0.5% NP-40, 150 mM NaCl, and 20 mM MgCl_2_.6 H_2_O, pH 7.4. HA–Ub–PA protein complexes were immunoprecipitated using 150 μl of prewashed anti-HA agarose slurry overnight at 4 °C with end-over-end rotation. The agarose slurry was then washed four times, and the HA–Ub–PA protein complexes were eluted using 110 μl of 2× Laemmli buffer. To check for efficient immunoprecipitation, 10 μl of the eluates were run on a Western blot.

The remaining 100 μl of the eluates were reduced with 20 mM DTT for 10 min at 95 °C and alkylated with 40 mM of iodoacetamide for 30 min at room temperature in the dark. Proteins were then acidified to 1.2% phosphoric acid, diluted sixfold with 90% methanol/100 mM tetraethylammonium bromide, and captured/washed on an S-trap column according to the standard protocol (27). Proteins were digested on the S-trap column with 2 μg of trypsin overnight at 37 °C. Eluted peptides were then dried and resuspended in 2% acetonitrile (ACN) and 0.1% formic acid (FA).

##### LC–MS/MS

Peptides were analyzed using a Dionex Ultimate 3000 nano-ultra high pressure reversed-phase chromatography system coupled on-line to an Orbitrap Fusion Lumos mass spectrometer (Thermo Scientific). Samples were separated on an EASY-Spray PepMap RSLC C18 column (500 mm × 75 μm, 2 μm particle size; Thermo Scientific) over a 60 min gradient of 2 to 35% ACN in 5% DMSO, 0.1% FA, and at 250 nl/min. The column temperature was maintained at 50 °C with the aid of a column oven. The mass spectrometer was operated in positive polarity mode with a capillary temperature of 305 °C. DIA mode was utilized for automated switching between MS and MS/MS acquisition as described previously (28). Briefly, full scans (*m/z* 350–1650) were acquired in the Orbitrap with 120 k resolution and maximum injection time of 20 ms, followed by 40 DIA scan windows with variable widths (supplemental Table S1). MS/MS fragmentation was performed in the higher energy collisional dissociation cell with a collision energy set at 30%. MS2 scans were acquired in the Orbitrap between *m/z* 200 and 2000 at a resolution of 30 k. The minimum points-per-peak was enabled and set to 6 with a dynamic maximum injection. All data were acquired in profile mode.

##### DIA–MS Data Processing and Analysis

Data were analyzed using DIA-NN (version 1.8) with all settings as default (29). Specifically, we allowed for a maximum of one tryptic missed cleavage and fixed modifications of N-term M excision and carbamidomethylation of Cys residues. No variable modifications were selected. A *Homo sapiens* UniProt database (20,370 entries, retrieved on April 16, 2021) was used for the analysis. A default threshold of 1% false discovery rate was used at the peptide and protein levels. We used the library-free mode of DIA-NN to generate precursor and corresponding fragment ions *in silico* from the UniProt database (29). The software also generates a library of decoy precursors (negative controls). Retention time alignment was performed using endogenous peptides, and peak scores were calculated by comparison of peak properties between observed and reference spectra. Immunoprecipitations were carried out in duplicate, and any DUBs that were not present in both control samples and enriched greater than fivefold when compared with a no probe control were discarded from the analysis. Identifications that were assigned to multiple DUBs were not included in the analysis. MINDY3 was also removed from the dataset as it was of the lowest intensity, so may have been at the bottom of the instrument's dynamic range and did not produce stable values across the dataset. Duplicate measurements were acquired for all samples and used to determine experimental statistics. All MS raw files were deposited in PRIDE under the code PXD036574.

#### Enzyme Kinetics

##### *In Vitro* USP30 Activity Assay

Fluorescence intensity measurements were used to monitor the cleavage of a Ub–rhodamine (Ub-Rho110) substrate. All activity assays were performed in black 384-well plates in 20 mM Tris–HCl, pH 8.0, 150 mM potassium glutamate, 0.1 mM Tris(2-carboxyethyl)phosphine) (TCEP), and 0.03% bovine gamma globulin with a final assay volume of 20 μl. Compound IC_50_ values for DUB inhibition were determined as previously described (18). Briefly, an 11-point dilution series of compounds were dispensed into black 384-well plates using an Echo-550 Acoustic Liquid Handler (Beckman Coulter). USP30, 0.2 nM (residues 64–502Δ179–216 & 288–305; Viva Biotech [Shanghai] Ltd), was added, and the plates were preincubated for 30 min, 25 nM Ub-Rho110 (Ubiquigent) was added to initiate the reaction, and the fluorescence intensity was recorded for 30 min on a PHERAstar FSX (λ_Ex_ = 485 nm, λ_Em_ = 520 nm) (BMG Labtech). Initial rates were plotted against compound concentration to determine IC_50_. Data were processed using analysis tools from Dotmatics (https://www.dotmatics.com/). RapidFire MS coupled to an Agilent QTof 6530 mass spectrometer (30) was used to confirm USP30 complex formation with USP30_inh_ and assess cleavage of K6-linked di-Ub chains from USP30 in the presence and absence of the compound.

##### Kinetic Assays–Determination of Kinetic Parameters for Slow-Tight Binding Inhibitors

Kinetic assays were performed in 384-well SensoPlate in 20 mM Tris–HCl, pH 8.0, 300 mM potassium glutamate, 0.1 mM TCEP, and 0.2% BGG with a final assay volume of 50 μl. An 11-point dilution series of compound was dispensed into assay plates, and 25 μl 2X Ub-Rho110 was added. The dispense function of the FLIPR Tetra (Molecular Devices) was used to add 25 μl 2X USP30 to give final assay concentrations of 5 and 180 nM for USP30 and Ub-Rho110, respectively. The fluorescence signal of the enzyme activity was monitored every 3 s for 1800 s (λ_Ex_ = 470–495 nm, λ_Em_ = 515–575 nm, camera gain 70, exposure time 0.6 s, and excitation intensity 80%). Analysis was performed in GraphPad Prism, version 9.4.1 for Windows (GraphPad Software; www.graphpad.com). The time course data were normalized relative to enzyme in the absence of compound and used to generate inhibition curves at each time point.

**Fig. 1:**
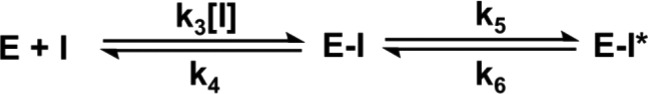
**Schematic showing slow-tight inhibitor (I) binding to enzyme (E)**. This is a two-step process with the fast formation of a less stable intermediate complex (E-I) defined by k3 and k4, followed by the formation of a more stable but still reversible complex (E-I∗) defined by k5 and k6. When k6 approaches zero, the complex is essentially irreversible.

As IC_50_ values are time dependent for USP30_inh_ with no covalent labeling of USP30, shown by MS (supplemental Fig. S2), data were modeled to a slow-tight binding scheme (Fig. 1). Fitting of progress curves allows for calculation of relevant kinetic parameters (Supplemental Information).

##### Biolayer Interferometry

Biolayer interferometry was performed on an Octet RED384 system (Sartorius) at 25 °C in a buffer containing 20 mM Tris–HCl (pH 8), 100 mM NaCl, 2 mM TCEP, 0.05% Tween, and 1% DMSO. Biotinylated USP30 (residues 64–502Δ179–216 & 288–305; Viva Biotech [Shanghai] Ltd) was immobilized onto super streptavidin biosensors. After 60 s baseline detection, the association of defined concentrations of USP30_inh_ (0–5 μM) was recorded over 300 s followed by dissociation in buffer over 600 s. Traces were normalized by double subtraction of baseline (no compound) and reference sensors (no USP30, association, and dissociation of compound) to correct for nonspecific binding to the sensors. Traces were analyzed using the Octet software (version 11.2; Sartorius).

#### HDX-MS

##### HDX Sample Preparation

USP30 was incubated in either the presence (holo-USP30) or absence (apo-USP30) of a twofold molar excess of USP30_inh_, ensuring that all complexes were fully formed and maintained over the course of the labeling reaction. Before the HDX-MS experiments, labelling (L), equilibration (E), and quench (Q) buffers were freshly prepared with D_2_O or H_2_O, respectively (buffers E and L: 50 mM Hepes, 400 mM NaCl, 2.0 mM TCEP, 10% glycerol [v/v] at pH 7.2; buffer Q: 50 mM potassium phosphate buffer, 2.0 M guanidine hydrochloride at pH 2.30). The USP30 protein sample was supplied at 66 μM and was diluted in buffer E to a final concentration of 11 μM, which equates to 16 pmol injected onto the pepsin column. Buffers E and L were equilibrated at 20 °C, whereas the protein samples and buffer Q were equilibrated at 0 °C.

##### HDX Cyclic Ion Mobility Mass Spectrometry

HDX-MS experiments were carried out on a fully automated HDX-2 system (supplied by Waters and previously described by Brown and Wilson (31). The exchange reaction was initiated by diluting 3.5 μl protein sample with a concentration of 11 μM into 56.5 μl buffer E for reference, or buffer L for D_2_O labeling, and incubated for several time points (0, 30, 60, 600, and 3600 s). A D_2_O/H_2_O ratio in excess of 90% guaranteed that the kinetics favored unidirectional exchange. Subsequently, the exchange reaction was stopped by mixing 50 μl of sample with 50 μl precooled buffer Q. Next, 50 μl of quenched sample was subjected to a temperature-controlled chromatography system (HDX M-Class UPLC; Waters). The protein was digested online by a pepsin column (Enzymate BEH pepsin column; 2.1 × 30 mm; Waters). Eluting peptides were trapped and washed on a C18 precolumn (C18 1.7 μM VanGuard 2.1 × 5 mm precolumn; Waters) at 100 μl/min for 3 min and separated on a reversed-phase column (C18 1.7 μM Acquity UPLC 1 × 100 mm reverse-phased column; Waters) with a linear gradient ranging from 5% ACN to 40% ACN plus 0.2% FA at 40 μl/min in 8 min, followed by a rapid rise to 99% ACN and holding for 0.3 min. ACN concentration was rapidly reduced to 5% and held there for 0.2 min, followed by a linear gradient back to 99% over 0.7 min, and holding that concentration for 0.1 min. Next, C18 columns were equilibrated with 95% H_2_O plus 0.2% FA for 4 min. The reversed-phase chromatographic system was kept at approximately 0 °C to reduce back-exchange. Peptides eluting from reversed-phase column were measured with a SELECT SERIES Cyclic IMS mass spectrometer (Waters) in HDMS^E^ mode (*m/z* 50–2000). This mode utilizes ion mobility separation for orthogonal separation of the peptides (LC, ion mobility, *m/z*). The mass spectrometer was fitted with an electrospray source equipped with additional independent LockSpray probe (Leu–enkephalin lock mass solution was used, *m/z* 556.2771).

##### HDX-MS Data Processing and Analysis

All MS analyses were performed in triplicate for each time point and condition. Protein Lynx Global Server 3.0 (Waters Corporation) was used for peptide identifications. A peptic peptide sequence coverage map was generated in DynamX 3.0 HDX software (Waters Corporation). Peptide-level deuterium uptake data were also visualized in DynamX and reported as relative deuterium exchange levels expressed in either mass unit or fractional exchange. The latter was calculated by dividing the experimentally measured uptake by the theoretically maximum number of exchangeable backbone amide hydrogens that could be replaced within each peptide. This number corresponds to the number of amino acid residues present in the peptide minus the number of proline residues and minus one for the N terminus that back exchanges too rapidly to be measured by MS (32). A single charge state was considered per peptide. Data were also verified and visualized in MEMHDX (33), which uses a mixed-effects model for HDX-MS statistical validation, factoring in the time dependency of the HDX reaction and number of independent replicates. The software generates two adjusted *p* values for each peptide, the first for the “change in dynamics” and the second for the “magnitude of ΔD.” These *p* values were subsequently used to categorize the data by means of a “Logit” plot (not shown). HDX-MS raw files were deposited in PRIDE under the unique identifier PXD041582.

#### Molecular Docking

The crystal structure of human USP30 catalytic domain (residues K64-V502) in covalent complex with Ub–PA at 2.34 Å resolution represents the highest resolution human USP30 structure available in the Protein Data Bank ([PDB] code: 5OHK) (11) and was used as the target receptor for docking the selective USP30 benzosulfonamide inhibitor, compound USP30_inh_ (20), using AutoDock Vina implemented in the program AMDock 1.5.2 (34). Coordinates for USP30_inh_ were generated using ChemDraw Prime 16.0.1.4 and PRODRG implemented in the CCP4 software suite (35). The compound was docked using the simple docking mode and automatic defined search space in AMDock 1.5.2.

## Results and Discussion

### USP30_inh_ is Highly Potent and Selective for Neuronal USP30

**Fig. 2:**
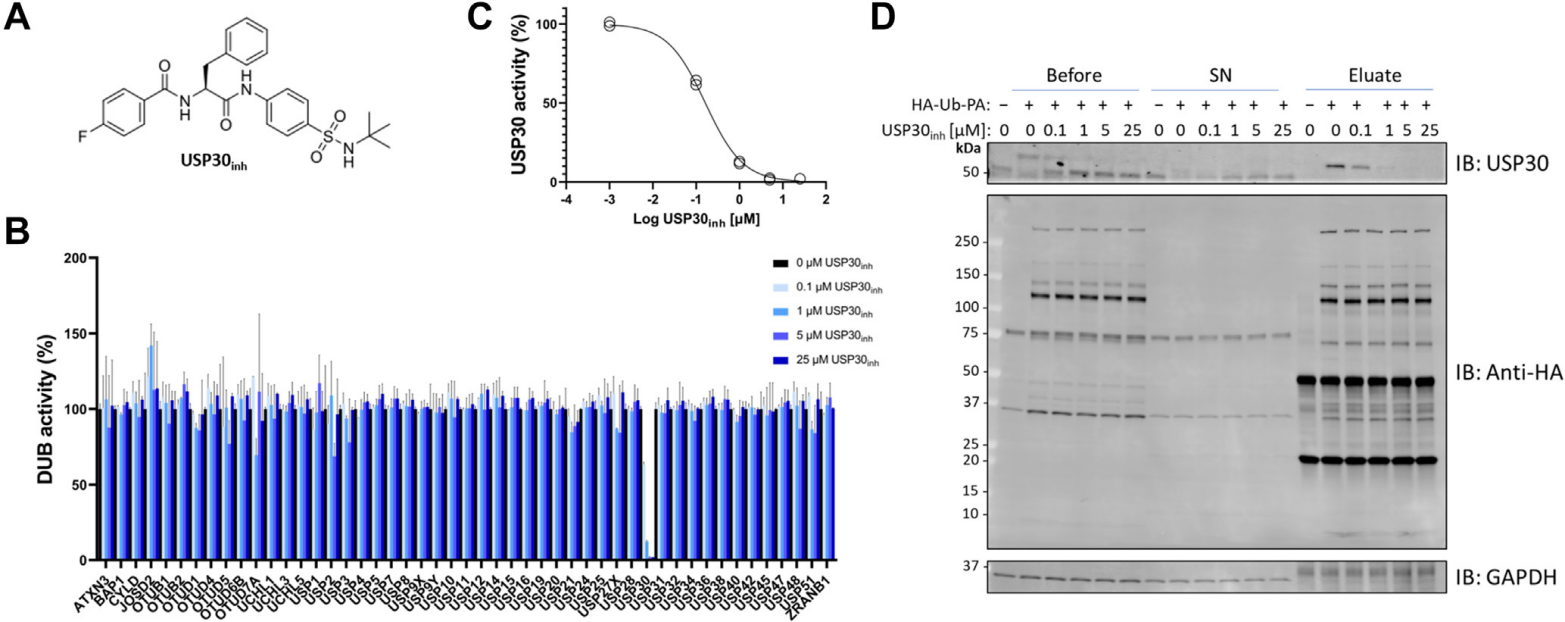
**USP30**_**i**__**nh**_**is highly potent and selective for USP30 inhibition in a cellular context.***A*, chemical structure of USP30_inh_ (25). *B*, activity-based probe profiling of cysteine-reactive deubiquitinating enzymes (DUBs) with a hemagglutinin-tagged ubiquitin-based propargylamine warhead probe (HA–Ub–PA). SH-SY5Y lysates were incubated first with USP30_inh_ at the indicated concentration for 1 h at 37 °C, followed by HA–Ub–PA for 45 min at 37 °C. DUB-probe complexes were immunoprecipitated by the HA tag and quantified by label-free LC–MS/MS. DUB intensity in the control with no USP30_inh_ present is normalized as 100% activity. n = 2 for each condition. *C*, USP30 intensity values extracted from (*B*), demonstrating that USP30_inh_ behaves in a concentration-dependent manner, with an IC_50_ of 0.16 μM. *D*, Western blot corresponding to data in (*B* and *C*). Immunoprecipitation of USP30 is reduced with increasing concentration of USP30_inh_. Anti-HA blot demonstrates efficient immunoprecipitation, and no USP30_inh_ cross-reactivity with other HA–Ub–PA-labeled DUBS, in agreement with LC–MS/MS data in (*B*). USP30, ubiquitin-specific protease 30.

The efficacy and selectivity of USP30_inh_ across a panel of cysteine-active DUBs was initially screened in SH-SY5Y neuroblastoma cell lysates by ABPP (Fig. 2*A*). SH-SY5Y cell extracts were treated with a range of inhibitor concentrations from 0.1 to 25 μM, followed by incubation with an HA-tagged Ub-based probe with a PA warhead (HA–Ub–PA). DUB-probe complexes were immunoprecipitated by way of their HA tag and quantified using label-free quantitation LC–MS/MS. We implemented a DIA MS regime to maximize the depth and reproducibility of the DUB profiling assay (supplemental Tables S1 and S2) (29, 36). The concentration-dependent competition between compound USP30_inh_ and HA–Ub–PA for binding to USP30 confirmed target engagement and potency of the inhibitor in a cellular matrix, with an IC_50_ value in the nanomolar range (Fig. 2*B*). Moreover, USP30_inh_ was found to be highly selective for USP30 as it had no significant activity against any of the other 49 endogenous DUBs detected in the experiment (Fig. 2*B*). The main cysteine-reactive DUB enzyme families were all represented, with proteins containing USP, ovarian tumor protease (OTU), Ub C-terminal hydrolase, and Josephin domains quantified (37, 38). The absence of USP30_inh_ concentration-dependent inhibition for all other identified cysteine-active DUBs demonstrates the highly selective nature of the inhibitor. This selectivity is in line with previously published USP30 inhibitor selectivity data from both a recombinant DUB activity panel (21) and an ABPP-MS experiment on a smaller panel of endogenous DUBs identified from mouse brain tissue (25).

We have recently reported that USP30_inh_ can be displaced by HA–Ub–PA over long incubations (25). Accordingly, a small amount of displacement during the 45 min HA–Ub–PA incubation was anticipated. It is therefore expected that the IC_50_ value of 0.16 μM that we obtained from our ABPP-MS assay is likely higher than the absolute IC_50_ inhibition concentration (Fig. 2, *C* and *D*).

### USP30_inh_ Binds USP30 in a Slow and Tight Manner

**Fig. 3:**
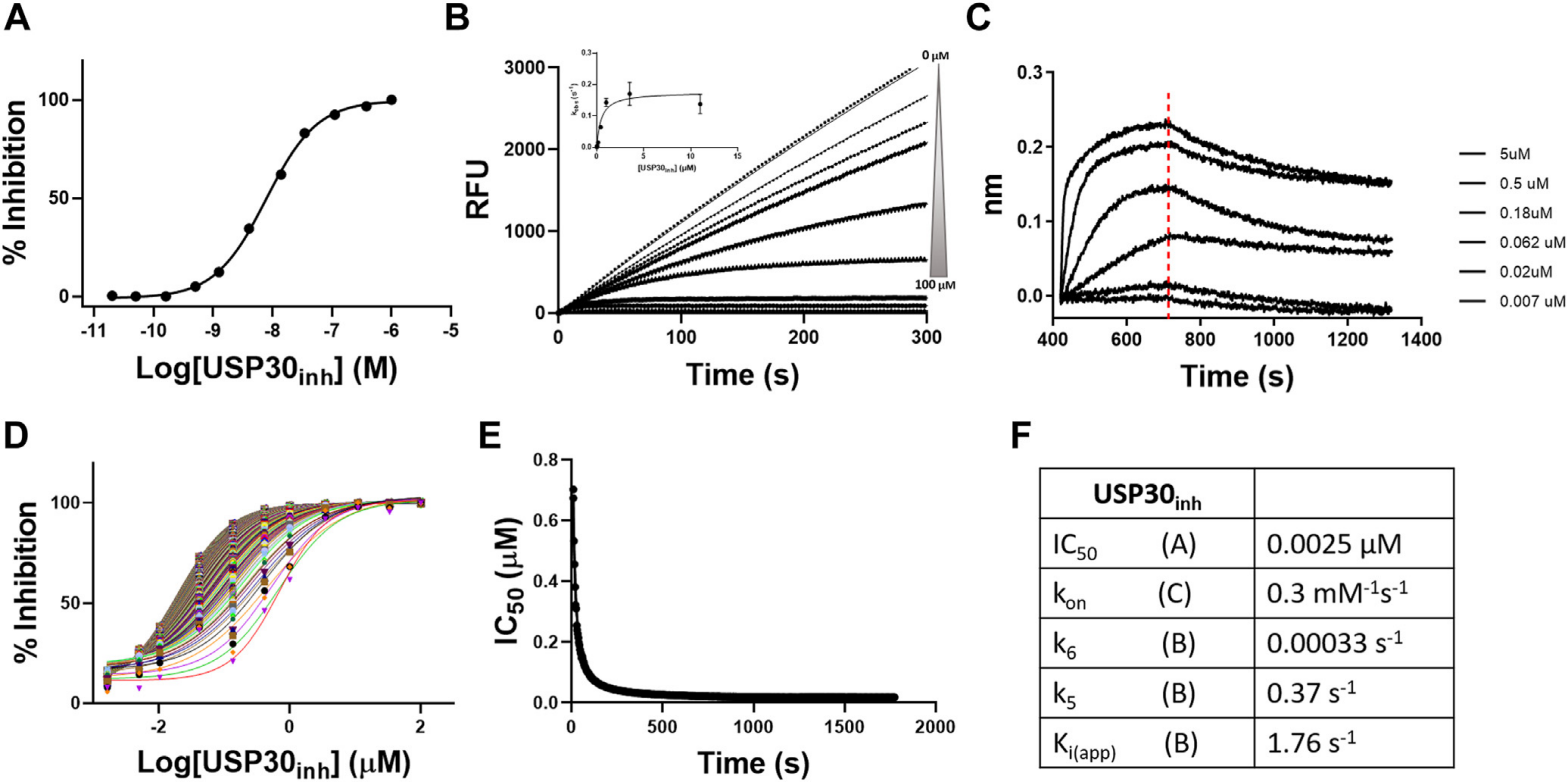
**Kinetic profiling of the noncovalent USP30 inhibitor USP30**_**inh**_**.***Upper panel*: *A,* dose-dependent inhibition of USP30 by USP30_inh_. *B*, reaction progress curves recorded on the FLIPR Tetra. Traditional method for determining kinetic constants associated with a two-step slow and tight binding inhibitor. *k*_obs_, determined by fitting the progress curves to Equation 2 (Supporting Information), is plotted *versus* [Compound] and fitted to Equation 3. (Supporting Information) to determine K_i_, k_5,_ and k_6_. *C*, biolayer interferometry showing binding of USP30_inh_ to immobilized USP30 with no detectable dissociation. *Lower panel*: (*D*) Krippendorf method (49) (Supporting Information) was used as an alternative way of determining kinetic constants. Time-dependent IC_50_ curves. Each curve represents inhibition data at an individual incubation time from 3 to 1800 s. *E*, IC_50_ values *versus* incubation time fitted to Equation 1 (Supporting Information) to obtain K_i_ and k_inact_. As USP30_inh_ is a noncovalent compound but has a k_6_ that is essentially 0, k_inact_ in this case represents k_5_. *F*, data table of inhibition properties. USP30, ubiquitin-specific protease 30.

Once it was established that USP30_inh_ downregulates endogenous USP30 activity in a highly selective fashion, we sought to rigorously profile its inhibitory properties using a recombinant version of the protein. Synthetic full-length USP30 is very unstable and difficult to solubilize (11). To circumvent this, we used a previously described truncated version of USP30 in our enzyme (and HDX-MS) assays, which readily went into solution and was determined to be stable over the time course of our experiments (supplemental Fig. S2). To assess enzyme deubiquitinating efficiency, the purified USP30 construct was incubated with a fluorogenic Ub–rhodamine substrate in both the presence and absence of USP30_inh_. This resulted in a calculated IC_50_ value for USP30_inh_ of ∼2 nM *in vitro*, which was in-line (albeit 10-fold lower) with previous estimations (Fig. 3*A*) (20). Although both measurements remain in the nanomolar range, the 2 nM IC_50_ from the Ub–rhodamine assay is lower than the 162 nM IC_50_ from the ABPP. This could be attributable to differences in the endogenous and recombinant activity of USP30, nonspecific inhibitor occlusion in the cellular context of the ABPP, or displacement of USP30_inh_ in the ABPP by HA–Ub–PA. Progress curves for Ub–rhodamine cleaved by USP30 were used to calculate the rate of inhibition. The kinetic constants k_5_, k_6_, and K_i_ gave values that were indicative of slow and tight binding behavior (Fig. 3, *B*, *D*, *E*, and *F*). The latter was visualized by a time-dependent shift of dose–response inhibition curves (Fig. 3*D*) as well as by plotting IC_50_ values against time (Fig. 3*E*). When considering the binding Figure 1 (Experimental Procedures section), the small value for k_6_ implies that it is behaving in an irreversible manner. The progress curves show typical features of an enzyme reaction in the presence of a slow binding inhibitor. Furthermore, two binding events are observed in the form of (a) an initial and (b) a steady-state velocity—both of which need to be considered during curve fitting and calculation of inhibitory rates (see Equations 2 and 3 in Supporting Information) (Fig. 3*B*).

Biolayer interferometry experiments confirmed this slow and tight binding behavior (Fig. 3*C*). The compound had an association rate of 0.3 mM^−1^ s^−1^ and a very slow dissociation from USP30 that was comparable with features of covalent modification (Fig. 3*F*). This phenomenon is intriguing, as USP30_inh_ is known to bind to USP30 by exclusively noncovalent means, as confirmed by our MS analysis in supplemental Fig. S2.

Finally, we explored the compound’s efficacy in the presence of more complex USP30–substrate interactions than Ub–rhodamine alone. We compared the cleavage of K6-linked di-Ub from USP30 in the presence and absence of USP30_inh_ by RapidFire MS (supplemental Fig. S3). It was clearly demonstrated that USP30_inh_ is able to inhibit the cleavage of K6-linked di-Ub from the substrate, confirming its activity in a more complex and physiologically relevant matrix.

### HDX-MS Kinetics Identifies Key Residues at the USP30_inh_-Binding Interface of USP30

Knowledge is currently lacking on the precise location and mechanistics of USP30_inh_ binding to USP30. HDX-MS experiments were consequently designed to pinpoint the key regions of USP30_inh_ binding to USP30, whilst providing novel structural insights into the solution conformation and dynamics of complex formation. HDX relies on the natural isotopic exchange of the amide backbone hydrogens of a protein with deuterium when placed in a deuterated solution (22). This leads to protein mass increases that are directly measurable by MS, which can serve as direct probes of protein solvent accessibility and structure. Shielding of the deuterated solvent following introduction of a binding partner is indicative of a binding interface. We sought to identify such regions following USP30_inh_ binding to USP30 by directly comparing the differences in HDX-MS uptake patterns of USP30 before (apo-USP30; in the presence of DMSO) and after (holo-USP30; in the presence of USP30_inh_) complex formation.

**Fig. 4:**
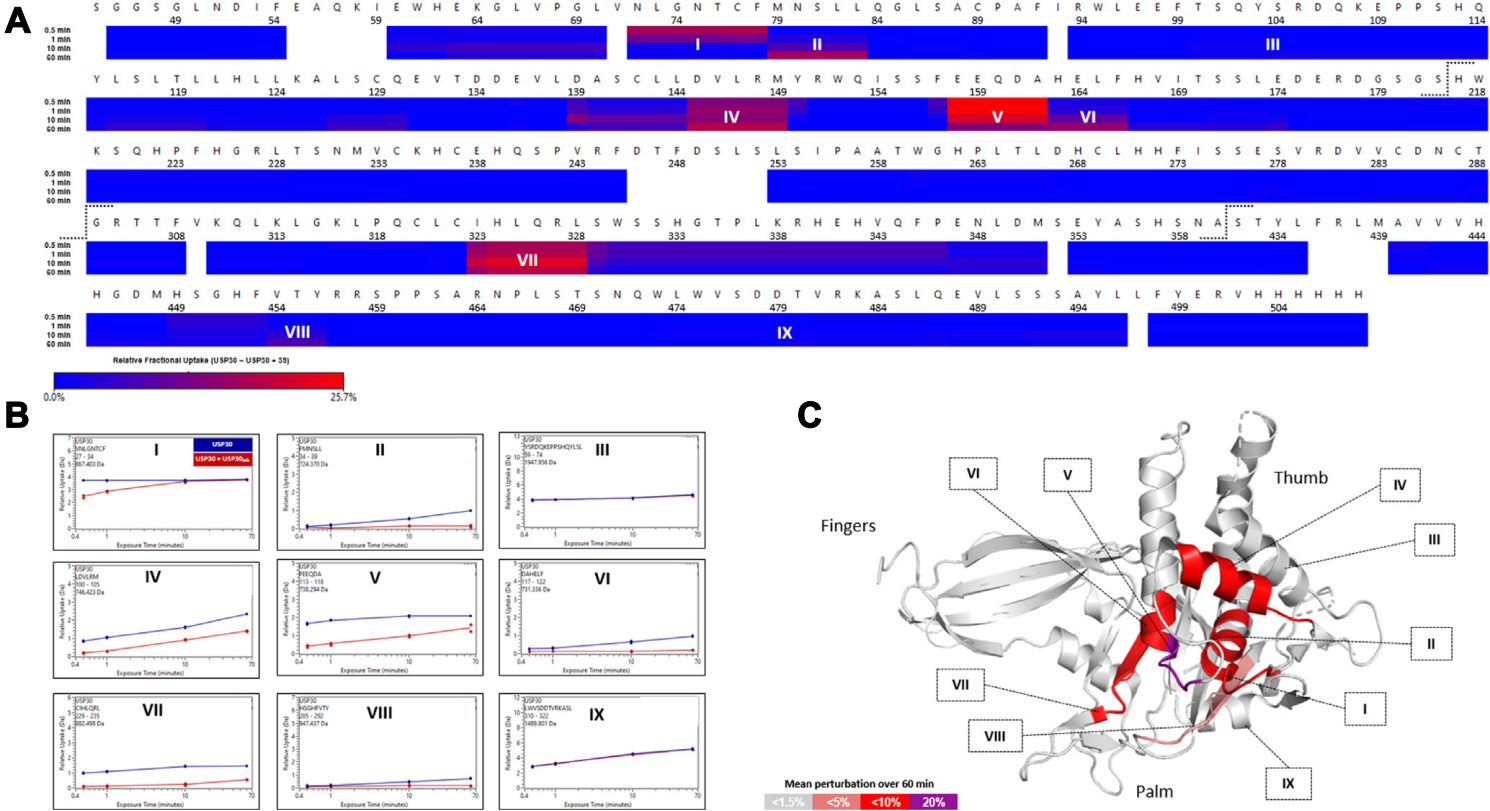
**HDX-MS characterizes the conformational dynamics of USP30**_**inh**_**binding to USP30.***A*, residue-level heat map indicating that USP30_inh_ induces solvent protection in several regions of USP30. The plot displays the difference in relative fractional uptake between the holo- and apo-form of the protein over 1 h. Regions of that have the greatest perturbation following USP30_inh_ binding are labeled regions I–IX. *B*, comparative uptake plots of regions I–IX for apo- and holo-USP30 states. *C*, integrated HDX-MS and X-ray crystal structure of USP30 in complex with di-Ub. Regions of perturbation between apo- and holo-USP30 states of HDX-MS data are colored according to magnitude of change. The data indicate that the USP30_inh_-binding interface is located between the USP30 thumb and palm domains of the protein. Numbering is in accordance with the crystal structure of 5OHK. *Dotted lines* indicate the site of cleavage and removal of unstable disordered sequences from the full-length USP30 protein. HDX-MS, Hydrogen–deuterium exchange mass spectrometry; Ub, ubiquitin; USP30, ubiquitin-specific protease 30.

Following digestion of unlabeled USP30 with pepsin, a total of 723 peptides were generated for the protein, 133 of which were shortlisted for downstream data analysis (supplemental Figs. S4 and S5). Selected peptides covered 96.2% of the USP30 sequence, with an average of 4.19 peptides covering each amino acid. The kinetics of deuterium uptake was analyzed for all regions of USP30, which included USP domains 1 to 6 and the catalytic triad at Cys77, His452, and Ser477 (supplemental Fig. S6*A*). From three independent replicates, the relative fractional exchange was calculated for all peptides at each of the four time points 30, 60, 600, and 3600 s, and plotted as a function of peptide position (Fig. 4*A* and supplemental Fig. S6*B*).

Following incubation with compound USP30_inh_ in conditions conducive to binary complex formation, our comparative HDX-MS data indicate that the majority of USP30 is unaffected by inhibitor binding, with no differences in deuterium uptake kinetics between apo and holo states (Fig. 4*A*). This suggests that USP30_inh_ binding is confined to smaller subsections of the protein. Indeed, several short regions of USP30 had significant shielding from the solvent in the presence of the inhibitor as compared with the DMSO control, indicative of regions involved in compound binding (Fig. 4, *A* and *B*). The areas of most significant perturbation included at two peptides spanning the USP30 catalytic Cys77 residue, N72–L83 (labeled regions I and II in Fig. 4 and highlighted in representative uptake plots in Fig. 4*B*), in addition to peptides mapping to D145–M149 (region IV), E158–F166 (regions V and VI), I323–L328 (region VII), and finally, H449–Y456 (region VIII), which encompasses the catalytic His452. These were in direct contrast to regions III and IX, which gave identical HDX-MS behaviors in both the apo and holo forms, reflecting the majority of the USP30 protein sequence (Fig. 4*B*). The shielding induced by inhibitor was greatest at the 1 h time point, where a difference of >25% was observed in the relative fractional uptake between states. Results were confirmed by the presence of multiple overlapping peptides displaying equivalent HDX-MS activity. Interestingly, no difference in conformational dynamics was observed between states for the region covering the catalytic Ser477 (region IX), suggesting that this site is not vital to the USP30_inh_ inhibitory process, but rather, may have a greater influence in determining USP30 Ub linkage preferences (11, 12, 39). Nevertheless, targeting this site to improve USP30 inhibition efficacies may prove fruitful in future design regimes.

No X-ray crystal structure of apo-USP30 or USP30 in complex with USP30_inh_ currently exists. We therefore mapped our solution HDX-MS data to PDB code 5OHK, which at a resolution of 2.34 Å, represents the highest resolution 3D structure of the protein currently available in the PDB (11). It is worth noting that this structure corresponds to USP30 in covalent complex with Lys6-linked di-Ub, and as such, may give rise to subtle discrepancies when comparing across results. Nevertheless, because of the lack of more suitable alternatives, we felt it a worthwhile pursuit to map our apo- and holo-USP30 HDX-MS data to this 3D model. To facilitate a facile overview of the entire dataset, HDX-MS results were collapsed into a single datapoint by calculating the mean perturbation between apo and holo states across the four labeling time points. USP30 constitutes three subdomains designated “thumb, palm, and fingers,” which is in line with related USP family members of elucidated structure (8, 40). Strikingly, the shielded USP30 peptides in the presence of USP30_inh_ all cluster to the same spatially adjacent region of the protein, which lies at the interface of its palm and thumb subdomains (Fig. 4*C*). Moreover, the regions with the greatest perturbation as highlighted previously, I323–L328 (region VII) and H449–Y456 (region VIII), cover areas of the protein that lie opposite to each other on the 3D structure. They may represent an entrance vector to the USP30-binding pocket, which is anticipated to be closer to the site of greatest perturbation at E158–F166 (regions V and VI) and the nearby catalytic Cys77. The importance of these residues to the inhibitory process is further strengthened by their correlation with the proposed region of USP30 binding to the Ub C-terminal tail in the crystal structure (11).

### Binding of USP30_inh_ Alters USP30 Conformation and Induces Rigidification in Several Regions

Although likely to be in good agreement with the majority of 5OHK, the structural makeup of apo-USP30 has yet to be experimentally confirmed. As yet, no high-resolution crystal structure exists, which is of a direct consequence of the poor stability of the full-length protein itself (11). As stated earlier, a highly truncated USP30 construct was used in this study, which was devoid of its N-terminal mitochondrial intermembrane domain and adjacent transmembrane domain. Furthermore, several long disordered regions were cleaved, and multiple hydrophobic residues mutated out, resulting in substantially improved protein stability and solubility. As HDX-MS is not reliant on successful crystallization trials, we saw this as an opportunity to describe the solution structural integrity of apo-USP30, which would allow us to elucidate its mode of binding to USP30_inh_.

The conformational landscape of apo-USP30 generally follows the arrangement of its USP domains; some of the most solvent-exposed regions of the protein are found at the linker regions connecting individual domains, most noticeably between USP domains 1 and 2, 4 and 5, and 5 and 6 (supplemental Fig. S6, *A* and *B*). Conversely, USP domains 1 and 5 and the N-terminal end of USP domain 6 are largely protected from the solvent and inaccessible (supplemental Fig. S6*B*). This is in good agreement with HDX-MS data recently acquired on the full-length apo-USP30 protein, where the USP domains were shown to be in a conformation that was generally hidden from the solvent and connected by several exposed linkers (11). However, because of the instability of the full-length species, HDX-MS was performed on a much shorter timescale compared with our own study, with only a sole 3 s labeling time point measured. Furthermore, an appreciation of USP30 dynamics could not be extracted from this single time point. Looking at apo-USP30 dynamics across the several labeling time points described herein, an increase in the rate of deuteration over the time course of the experiment was observed across the majority of the protein (supplemental Fig. S6*B*). This dynamic HDX-MS behavior is indicative of the presence of secondary structural elements, thereby confirming the highly structured nature of the protein. The regions with the greatest dynamic HDX-MS behavior were found within USP domains 2, 3, 4, and 6 (supplemental Fig. S6*B*). Conversely, no dynamic HDX-MS events were observed in several regions of apo-USP30, and the maximum level of deuteration was reached immediately, indicating structural disorder. These unstructured regions map to the N- and C-terminal extremities of the protein and within USP domains 1 (residues 71–78), 2 (residues 130–136), and 5 (residues 439–453). There is a high level of overlap when mapping the structural data inferred from the HDX-MS to the crystal structure of 5OHK for both apo- and holo-USP30 (supplemental Fig. S7).

Several regions of USP30 undergo structural transitions in the presence of USP30_inh_, which are potentially significant in terms of inhibitory mechanistics. First, multiple segments of USP30 become completely blocked and inaccessible to the solvent following inhibitor binding. These include a region directly adjacent to the catalytic Cys77 at F78–L83 and an area of USP domain 2 covering E158–F166, as highlighted in *purple* in supplemental Fig. S7. This suggests that USP30_inh_ induces a conformation of USP30 that not only blocks off the catalytic region and its surroundings from the solvent, but importantly, also prevents access and binding of Ub itself. A second structural phenomenon is also evident, specifically the conversion of intrinsically disordered loops in the absence of USP30_inh_, to rigid structural elements in the presence of the compound (supplemental Fig. S7). These disorder-to-order transitions likely embody functional significance (32), and in USP30, these are found at the catalytic Cys77, represented by peptide V71–F78, a section of USP domain 2 at R148–F154, and a long chain of residues spanning Q326–L349.

Tracking these structural rearrangements across individual labeling time points allows us to propose a general timeline of inhibition (supplemental Fig. S8). Taking Q326–L349 as an example, peptides mapping to this region of USP30 undergo significant structural transitions at the earliest time points monitored (30 and 60 s), which are completed in the later stages of our experimental time course. Conversely, peptides proximal to the catalytic Cys77 become blocked and solvent inaccessible primarily in the latter half of our experiment (600 and 3600 s). The fact that the residue (and adjacent regions) most crucial to USP30 catalysis, Cys77, is most significantly perturbed in the latter stages of our experiment could go some way to explain the slow and tight binding behavior observed for USP30_inh_ in our enzyme kinetics analyses (Fig. 3).

### Molecular Docking Proposes Key Residues Important for USP30_inh_ Binding to USP30

**Fig. 5:**
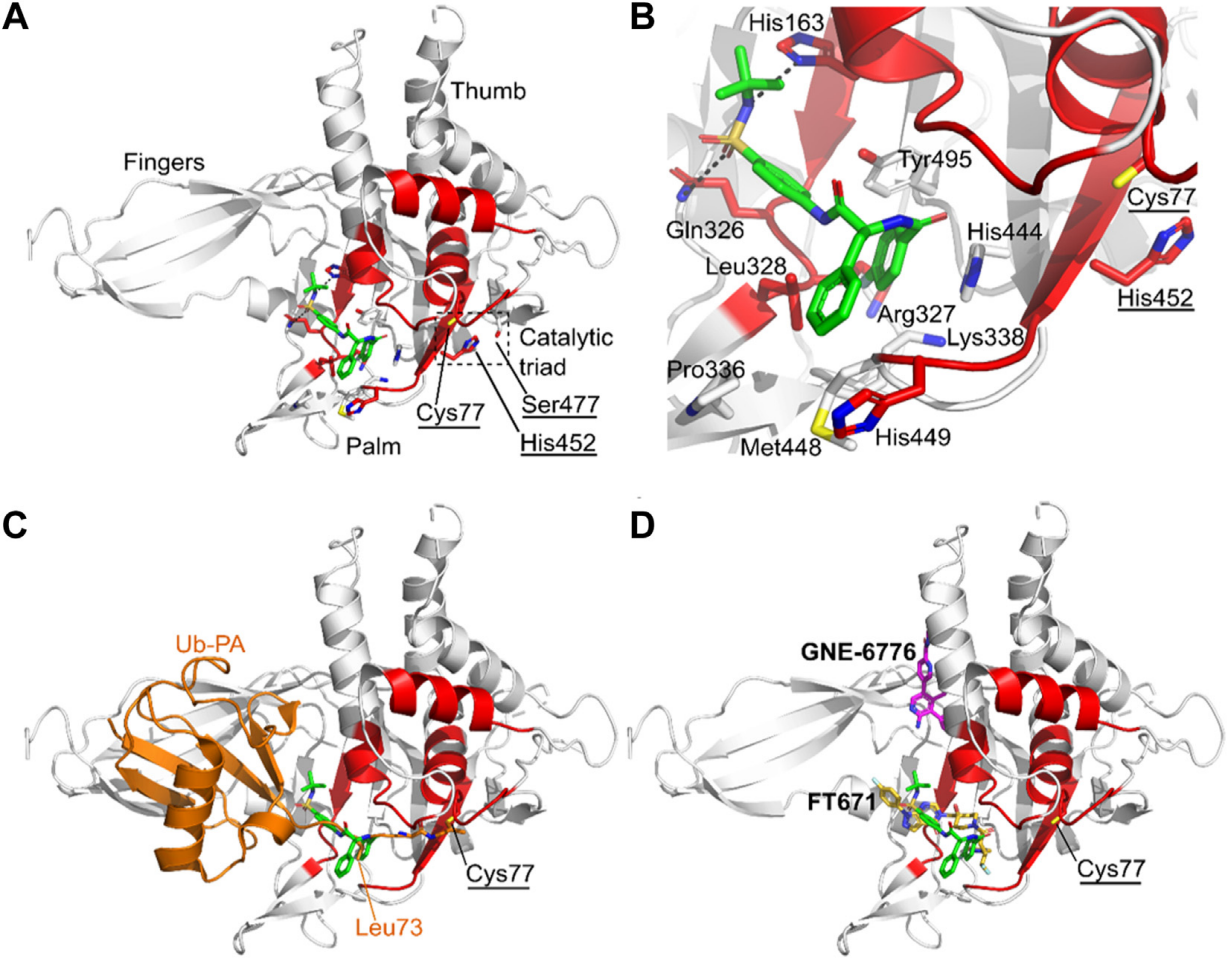
**Modeled structure of human USP30 in complex with USP30**_**inh**_**.***A*, structure of human USP30 catalytic domain highlighting the modeled position of USP30_inh_ shown as a *stick* representation and colored *green*. The thumb, palm, and finger's subdomains of the catalytic domain and catalytic triad (Cys77, Ser477, and His452; *underlined*) are highlighted. Regions identified in the HDX-MS analysis of USP30 in the presence of USP30_inh_ are colored *red*. *B*, close-up view of the putative USP30_inh_ binding site highlighting key residues and hydrogen-bonding interactions represented as *dotted lines*. *C*, superposition of Ub–PA (*orange*; PDB code: 5OHK) on the docked structure. USP30_inh_ sterically clashes with the C-terminal tail of the Ub substrate, thereby preventing Ub binding and isopeptide bond cleavage. *D*, superposition of the USP7 inhibitors, FT671 (*yellow carbon atoms*) and GNE-6776 (*magenta carbon atoms*) in complex with USP7, onto the docked structure. USP30_inh_ putatively binds to an equivalent site in the thumb–palm cleft compared with FT671. Figure was prepared using PyMOL (The PyMOL Molecular Graphics System, version 2.4.1; Schrödinger, LLC). PA, propargylamine; PDB, Protein Data Bank; Ub, ubiquitin; USP30, ubiquitin-specific protease 30.

To further refine our HDX-MS findings, we explored the binding mode of USP30_inh_ to USP30 computationally. We performed molecular docking simulations using the simple docking mode in AMDock software, with the human USP30 catalytic domain from the crystal structure of USP30 in complex with Ub–PA (PDB code: 5OHK) acting as the target receptor for the compound (11, 20, 34). The *in silico* binding pose of USP30_inh_ with the highest-ranking docking score has estimated affinity and K_i_ values of −7.9 kcal/mol and 1.62 μM, respectively, compared with a reported experimental IC_50_ value of approximately 20 nM (20). USP30_inh_ is predicted to bind to the thumb–palm cleft that guides the Ub C terminus into the active site, residing approximately 7.4 Å away at its closest point from the thiol side chain of catalytic Cys77 (Fig. 5*A*). The benzyl moiety of USP30_inh_ is flanked by Pro336, Met448, and the side chains of Leu328 and His449 (Fig. 5*B*). The fluorophenyl moiety is flanked by the side chains of Leu328, Arg327, Lys338, and Tyr495, with a π-stacking interaction with His444. The *N*-*tert*-butyl sulfonamide moiety is anchored by hydrogen-bonding interactions with the side chains of Gln326 and His163. Compared with the structure of USP30 in complex with Ub–PA (PDB code: 5OHK), the modeled position of USP30_inh_ would sterically clash with the C-terminal tail of the Ub substrate with the fluorophenyl moiety sitting in an equivalent position to the side chain of Ub Leu73, thereby preventing Ub binding and isopeptide bond cleavage (Fig. 5*C*).

Crystal structures of USP7 in complex with the small-molecule inhibitors, FT761 (PDB code: 5NGE) (40) and GNE-6776 (PDB code: 5UQX) (41), reveal two distinct inhibitor binding modes that attenuate Ub binding and inhibit the DUB activity. FT671 binds to the thumb–palm cleft and resides approximately 5 Å away from the catalytic cysteine, whereas GNE-6776 interacts with acidic residues in the USP7 catalytic domain that mediate hydrogen-bonding interactions with the Ub Lys48 side chain and binds 12 Å from the catalytic cysteine. A comparison of the docked structure of USP30_inh_ with the USP7 inhibitor complexes suggests that USP30_inh_ is most likely to bind to an equivalent site to FT671 (Fig. 5*D*). In addition, the docked pose of USP30_inh_ correlates well with the HDX-MS data, with the predicted binding site of USP30_inh_ being flanked by residues residing in peptides E158–F166, I323–L328, and H449–Y456, which become solvent protected in HDX-MS (Fig. 5, *A* and *B*). The HDX-MS analysis also implicates peptides N72–L83 (which contains the catalytic Cys77) and D139–M149 in structural rearrangements upon compound binding. These regions reside further from the predicted binding site of USP30_inh_. However, compound binding may potentially cause conformational rearrangements of the catalytic domain remote from the binding site resulting in these regions becoming protected upon compound binding. Similar conformational rearrangements are seen in human USP7, in which the inhibitors bind to the catalytically incompetent apoform state with the switching loop “in”, as compared with the catalytically competent Ub-bound state with the switching loop “out”. It is possible that USP30 may exhibit similar dynamic conformational flexibility.

### SAR of USP30 and USP30_inh_

The inhibitory activity of USP30_inh_ and a series of analogs were reported by Mitobridge Therapeutics (20). The SAR of this series indicates that the sulfonamide N–H hydrogen bond donor is essential for inhibitory activity since replacement of this with N–Me resulted in a complete loss of inhibition. This finding is consistent with the hydrogen-bonding interaction of the sulfonamide N–H with His163 shown by our predicted binding pose.

The reported SAR also showed that the two amide N–H groups are important for potency against USP30 indicating that they may form hydrogen-bonding interactions; however, the docking simulation did not find hydrogen-bonding interactions between these N–H groups and USP30. It may be that the predicted binding mode needs further refinement or that the methylated amide groups lose inhibitory activity against USP30 for other reasons such as a steric clash between the methyl groups and USP30.

The docking simulation also indicated that the benzyl group of USP30_inh_ contributes to binding by occupying a lipophilic pocket rather than forming π-stacking interactions. This is also consistent with the reported SAR, which indicates that the benzyl group can be replaced by other lipophilic groups, that is, a cyclohexylmethyl group. The 4-fluorophenyl group also occupies a lipophilic group in the predicted binding pose, and the docking simulation indicates that it can form a π-stacking interaction with His444. The reported SAR indicates that the 4-fluorophenyl group can be replaced both by other aromatic rings, which could maintain the π-stacking interaction (where the fluorine substituent is moved around the phenyl ring or where the phenyl ring is replaced with a pyridyl ring) or by a cyclohexyl ring that would not be able to form a π-bond indicating. This would suggest that any π-stacking interactions formed by the 4-fluorphenyl group are not essential for potent inhibition on USP30.

### Comparison of USP30_inh_ to Other Small-Molecule Compounds

Recently, the structures of human USP30 in complex with covalent inhibitors, **552** (PDB code: 8D1T; supplemental Fig. S9*A*) and **829** (PDB code: 8D0A; supplemental Fig. S10*A*), have been deposited in the PDB (https://www.lens.org/images/patent/WO/2020036940/A1/WO_2020_036940_A1.pdf). Comparisons with the structure of USP30 in complex with Ub–PA (supplemental Fig. S9, *B* and *C*; supplemental Fig. S10, *B* and *C*) reveal distinct differences in the conformation in blocking loops 1 and 2 and a switching loop region (residues 150–162), indicating that these regions are likely to be highly flexible and adopt different conformations upon Ub or compound binding. The covalent-inhibitor binding sites are flanked by regions implicated in noncovalent inhibitor USP30_inh_ binding from the HDX-MS analysis (supplemental Figs. S9*A* and S10*A*). Comparison with the docked structure of USP30 in complex with USP30_inh_ reveals that USP30_inh_ is likely to bind to a similar region in the thumb–palm cleft compared with **552** and **829** (supplemental Figs. S9*D* and S10*D*), with the benzyl moiety of USP30_inh_ overlaying on the cyclopropyl nitrile and cyclopropyl pyrazine moieties of **552** and **829**, respectively (supplemental Figs. S9*E* and S10*E*). Compared with **552** and **829**, the docked pose of USP30_inh_ extends toward the finger’s subdomain. It is conceivable that conformational flexibility in blocking loops 1 and 2 and the switching loop region, not accounted for in the modeling, may facilitate USP30_inh_ to bind closer to the catalytic cysteine, Cys77, compared with the docked pose.

## Conclusions

Mitochondrial pathway disruption has been linked to a spectrum of pathophysiological conditions, from neurodegeneration and acute, chronic kidney, and cardiovascular diseases, through to hepatocellular carcinoma and peroxisome biogenesis disorders (7, 42, 43, 44). USP30 represents an actionable drug target of these conditions through its participation in PINK1/Parkin-mediated mitophagy, BAX/BAK-dependent apoptosis, oncogenesis, and pexophagy (19, 45, 46, 47). USP30 regulates mitophagy by antagonizing Parkin-mediated ubiquitination, and its inhibition has been shown to have significant therapeutic potential against PD and similar neurodegenerative disorders. Drug discovery efforts targeting USP30 have yielded the highly potent and selective small-molecule benzosulfonamide inhibitor, compound USP30_inh_ (18, 20, 48). Combining state-of-the-art proteomics, HDX-MS and molecular docking, we have described the dynamic structural interplay between USP30 and USP30_inh_. The inhibitor binds to USP30 in a slow and tight manner, and displays kinetic properties consistent with covalent attachment to USP30, despite its noncovalent design. Collectively, our integrative structural biology lens successfully identified regions within USP30 that undergo dramatic structural and conformational rearrangements in the presence of USP30_inh_, which prevent Ub binding and decrease DUB activity. X-ray data for USP30 in complex with USP30_inh_ will undoubtedly complement these observations and will, combined with molecular dynamics studies, drive the development of next-generation inhibitors.

## Data Availability

The ABPP proteomics data files have been deposited to the ProteomeXchange Consortium and can be located under the PRIDE dataset identifier PXD036574. Annotated spectra ABPP data can be viewed following deposition to PanoramaWeb, which can be accessed using the ProteomeXchange ID code of PXD042782 and permanent link https://panoramaweb.org/LDvSvO.url. Similarly, the HDX-MS dataset can be located using the code PXD041582. PDB ID code 5OHK represents the crystal structure of human USP30 catalytic domain (residues K64–V502) in covalent complex with Ub–PA at 2.34 Å resolution. This is the highest resolution structure of human USP30 currently available in the PDB.

## Supplemental Data

This article contains supplemental data.

## Conflict of interest

The authors declare that they have no conflicts of interest with the contents of this article.
