# Supplementary material for: Structural Premise of Selective Deubiquitinase USP30 Inhibition by Small-Molecule Benzosulfonamides
Source: Mol Cell Proteomics. 2023 Jun 28;22(8):100609. doi: 10.1016/j.mcpro.2023.100609 (PMC10400906; doi:10.1016/j.mcpro.2023.100609)

Structural Premise of Selective Deubiquitinase USP30 Inhibition by Small-Molecule Benzosulfonamides

Darragh P O'Brien[1*], Hannah BL Jones[1], Franziska Guenther[2], Emma J Murphy[2], Katherine S England[2], Iolanda Vendrell[1], Malcolm Anderson[3], Paul Brennan[2], John B Davis[2], Adán Pinto-Fernández[1,4], Andrew P Turnbull[5], and Benedikt M Kessler[1,4*]

[1]Target Discovery Institute, Centre for Medicines Discovery, Nuffield Department of Medicine, University of Oxford, UK

[2]ARUK-Oxford Drug Discovery Institute, Centre for Medicines Discovery, Nuffield Department of Medicine, University of Oxford, UK

[3]Waters Corporation, Wilmslow, Cheshire, UK

[4]Chinese Academy of Medical Sciences Oxford Institute, Nuffield Department of Medicine, University of Oxford, UK

[5]Cancer Research Horizons, Francis Crick Institute, London, UK

*Corresponding authors: darragh.obrien@ndm.ox.ac.uk; benedikt.kessler@ndm.ox.ac.uk

SUPPLEMENTAL FIGURES

Contents

Figure S1: Purity profile of $USP30_{inh}$
Figure S2: Confirmation of USP30 complex formation with $USP30_{inh}$
Figure S3: Inhibition of USP30 cleavage of K6-linked Di-Ubiquitin by $USP30_{inh}$ as measured by RapidFire MS
Figure S4: Peptide map of USP30
Figure S5: HDX-MS uptake curves for individual USP30 peptides
Figure S6: Differential HDX-MS of USP30 and $USP30_{inh}$
Figure S7: Architecture of USP30 inferred by HDX-MS
Figure S8: Temporal exchange of USP30
Figure S9: Structure of human USP30 in complex with the covalent inhibitor, 552
Figure S10: Structure of human USP30 in complex with the covalent inhibitor, 829

Supplemental Figure 1

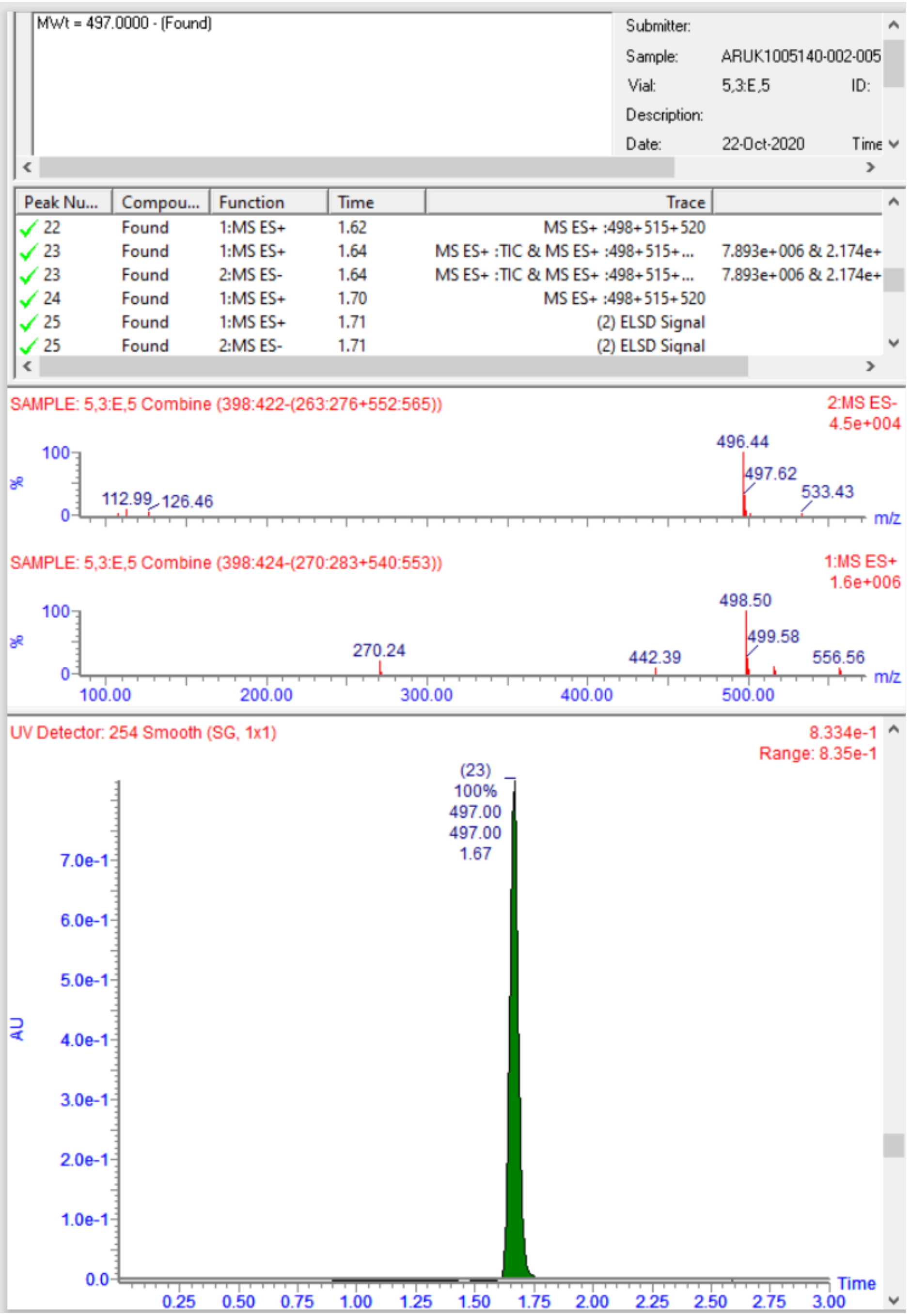

Supplemental Figure 2

1:1 USP30:USP30$_{inh}$

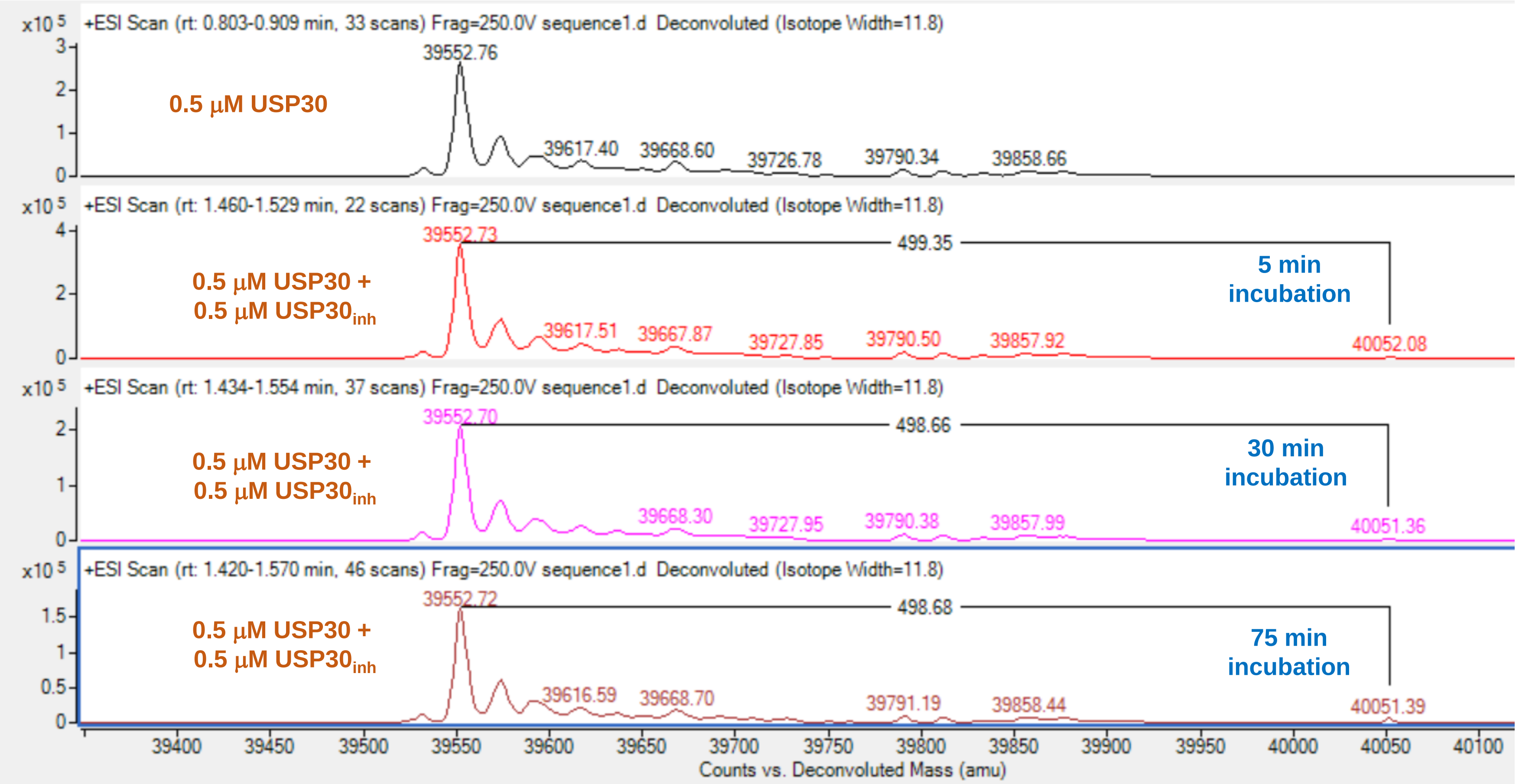

USP30$_{inh}$

MW: 497.6

USP30 (Viva)

- MW: **39552.24** Da
- insertion/deletion mutant: (64-178)-GSGS-(217-288)-G-(305-357) N346P, F348N, M350D, D352S, I353E, K355A, Y356S-SNA-(432-502)-6*His + biotin tag

Supplemental Figure 3

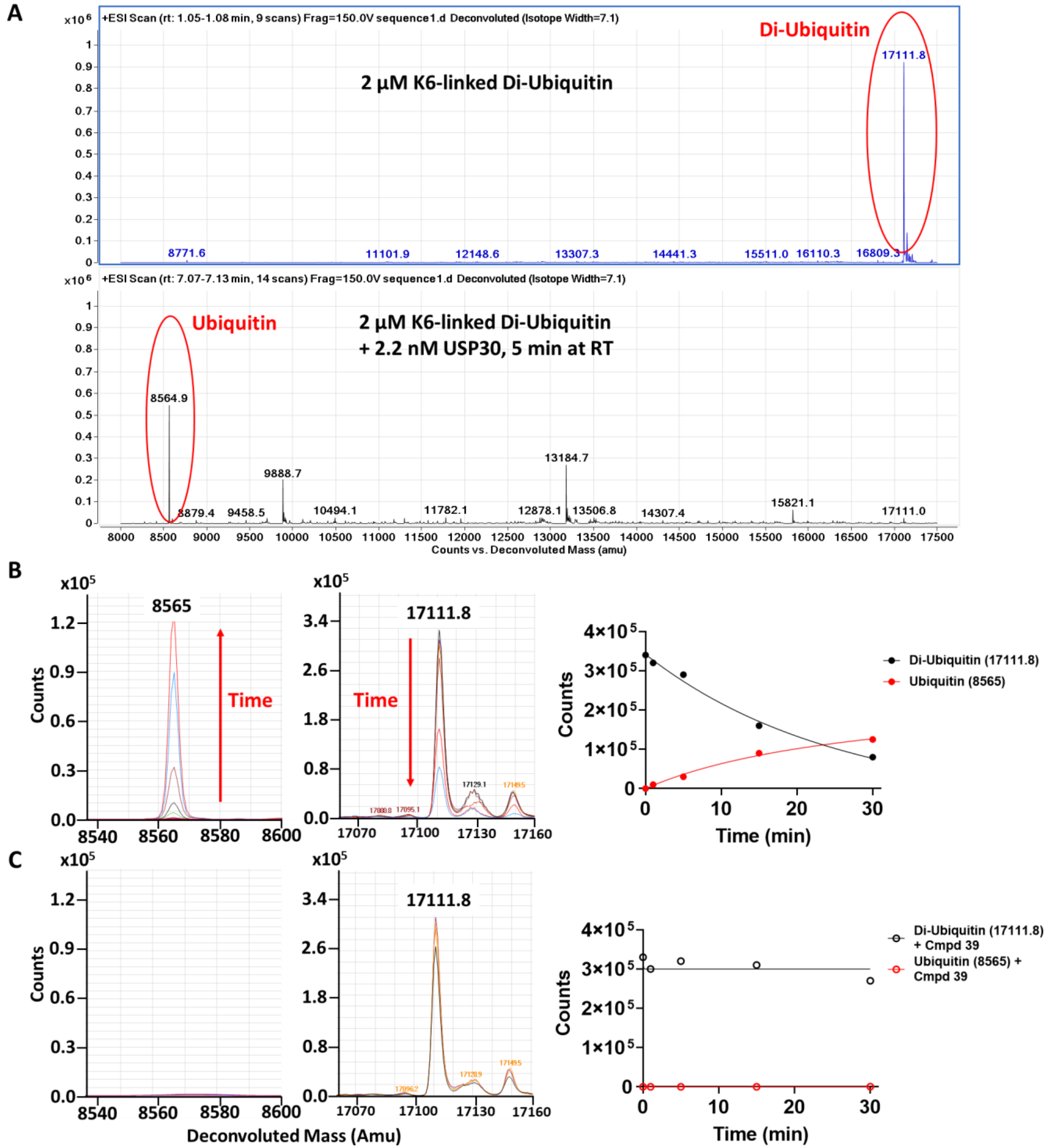

Supplemental Figure 4

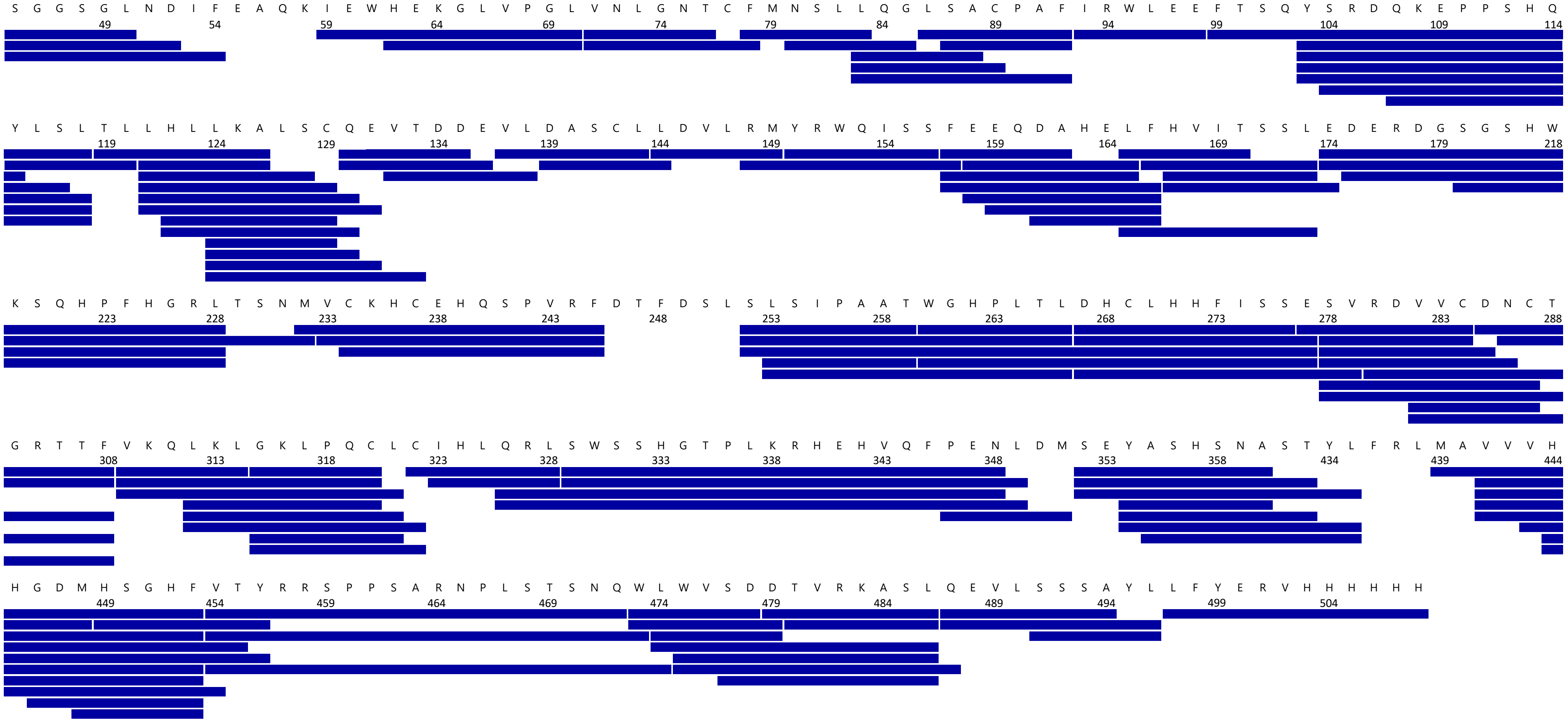

Supplemental Figure 5

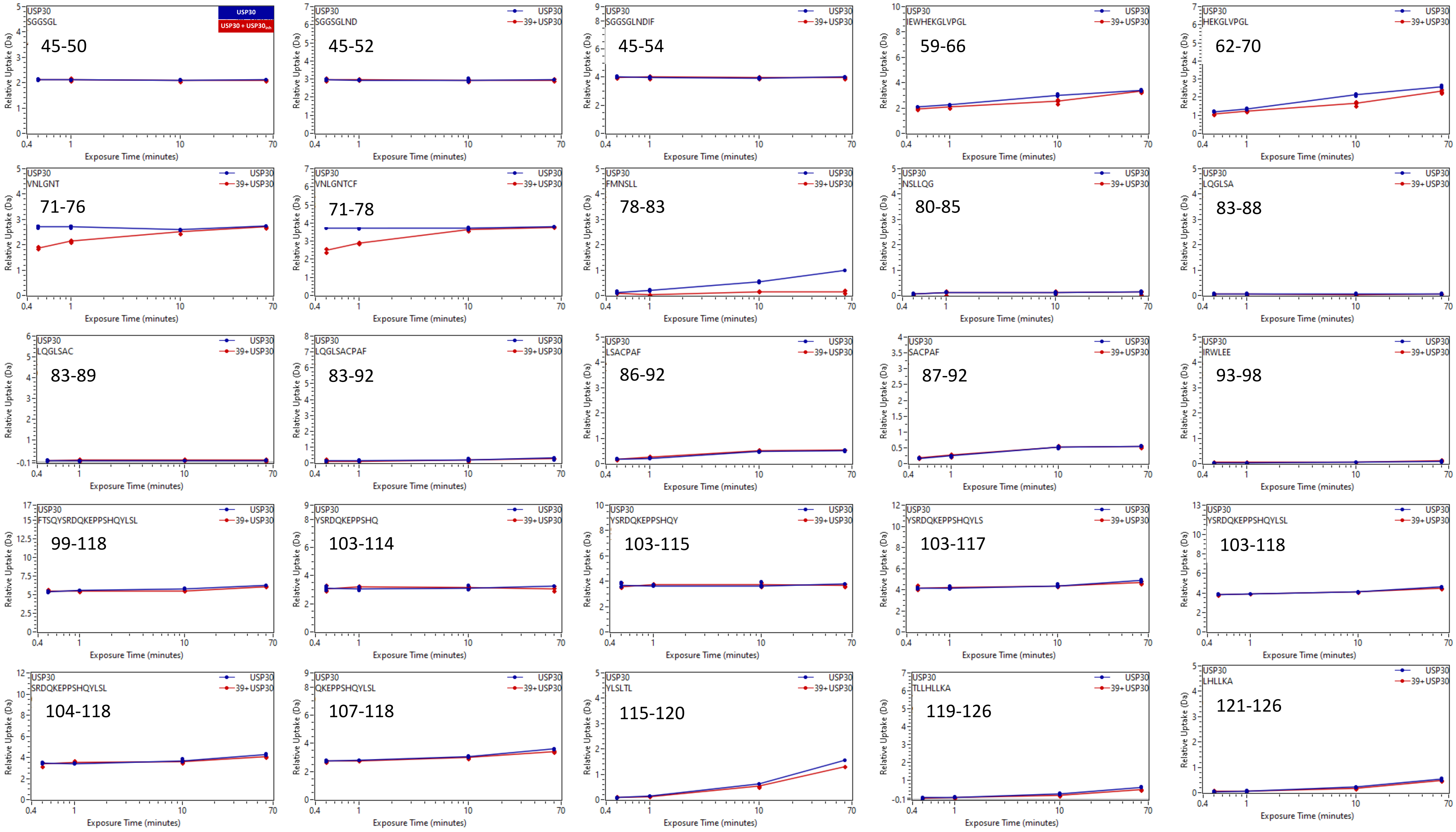

Supplemental Figure 5 (continued)

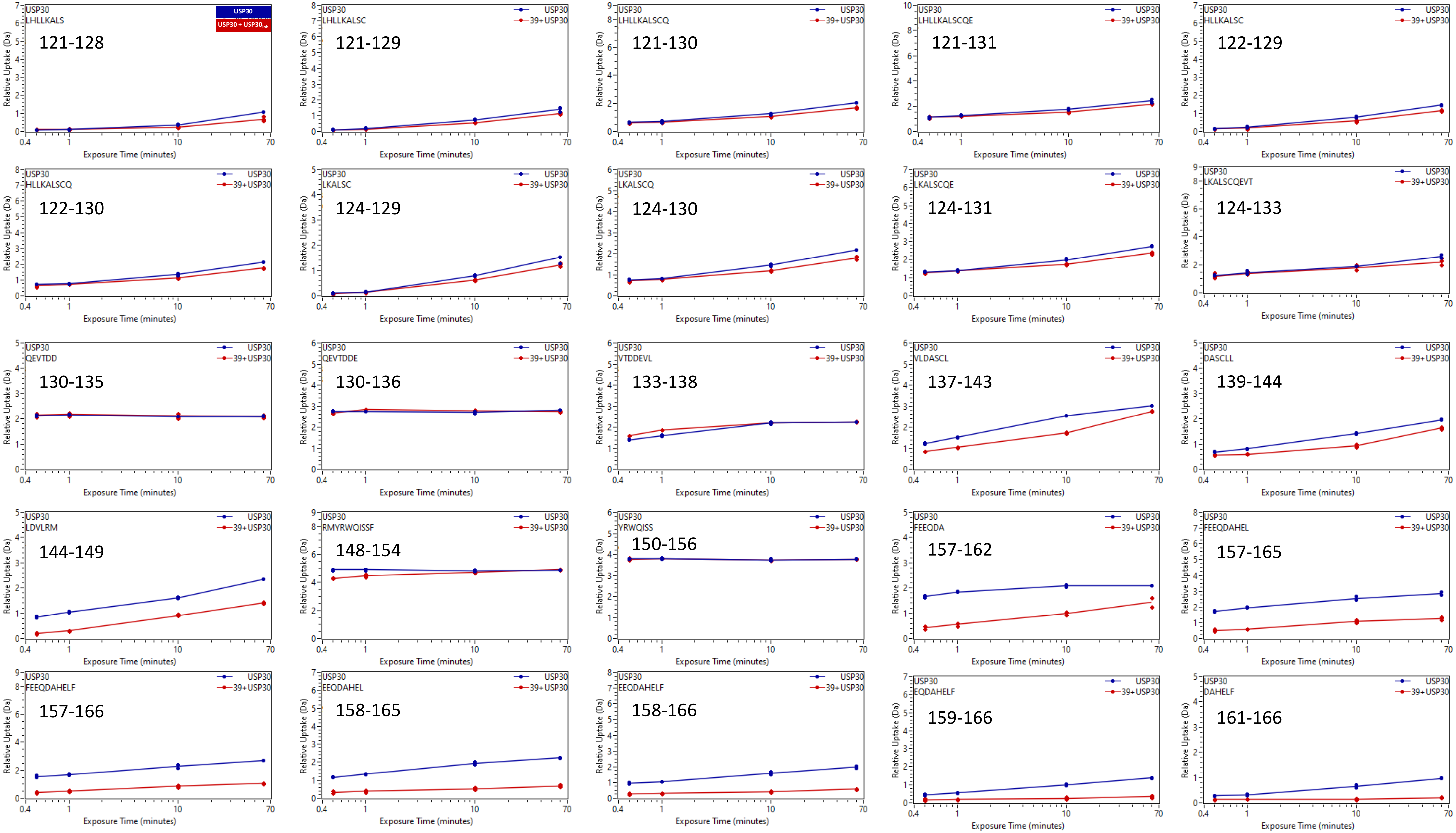

Supplemental Figure 5 (continued)

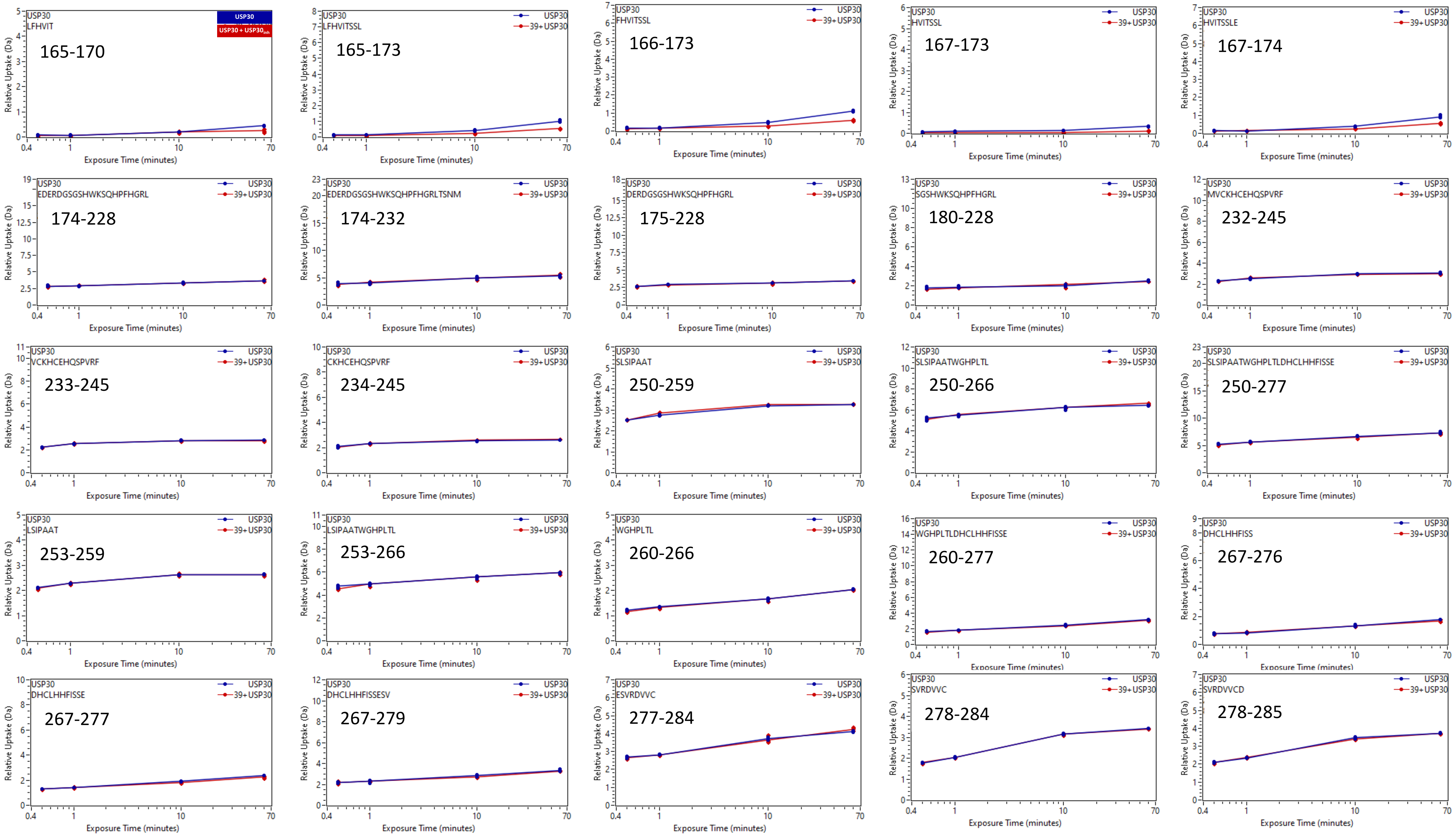

Supplemental Figure 5 (continued)

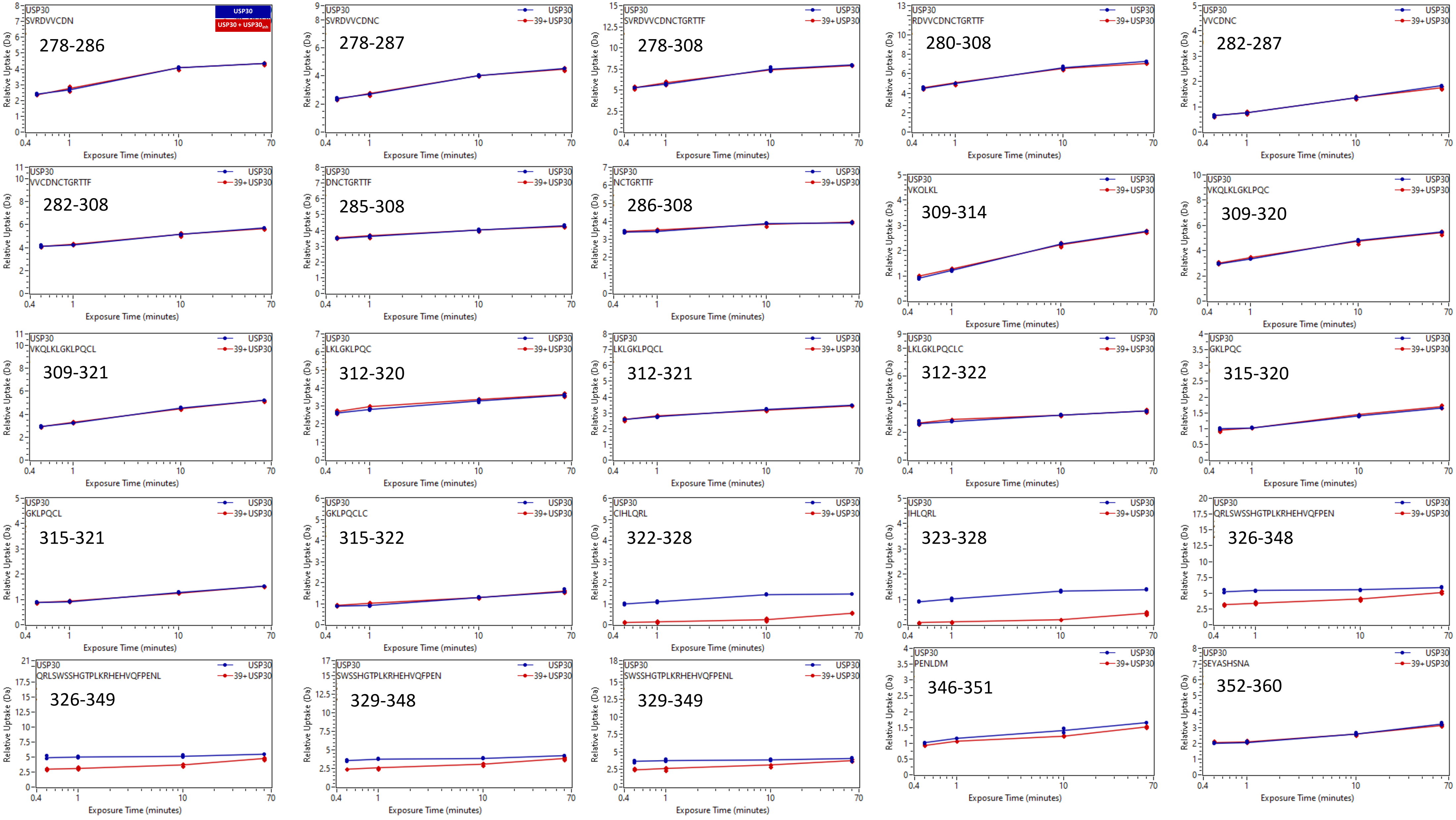

Supplemental Figure 5 (continued)

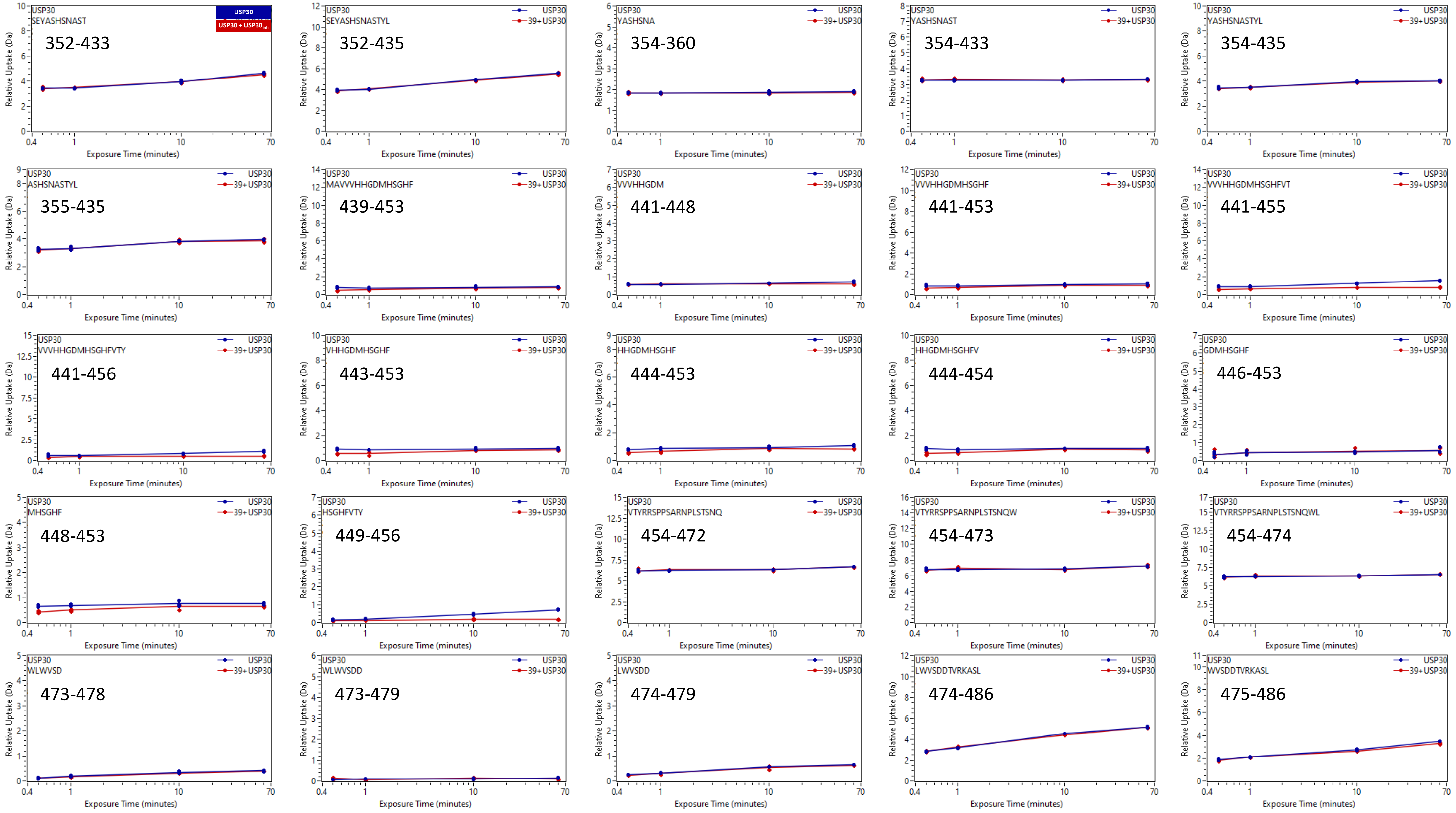

Supplemental Figure 5 (continued)

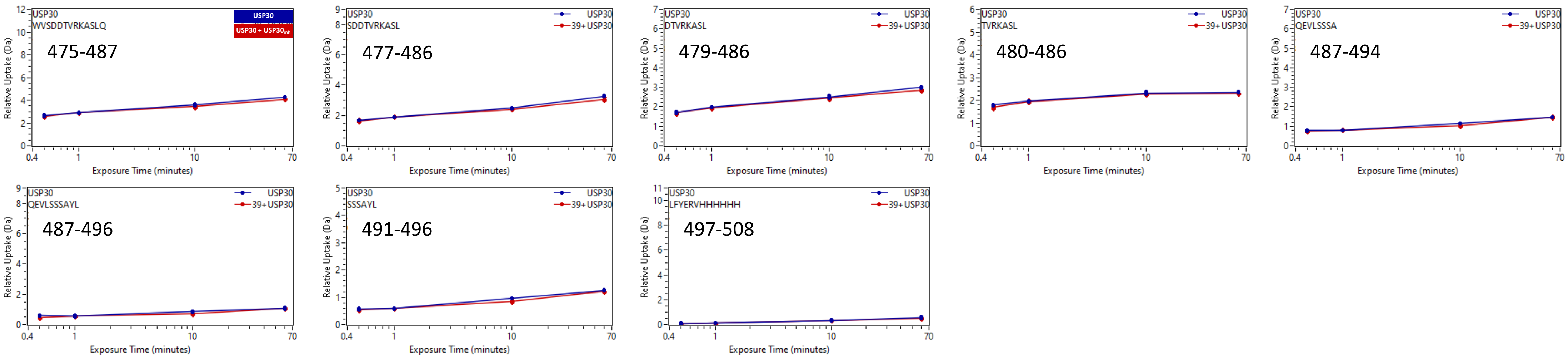

Supplemental Figure 6

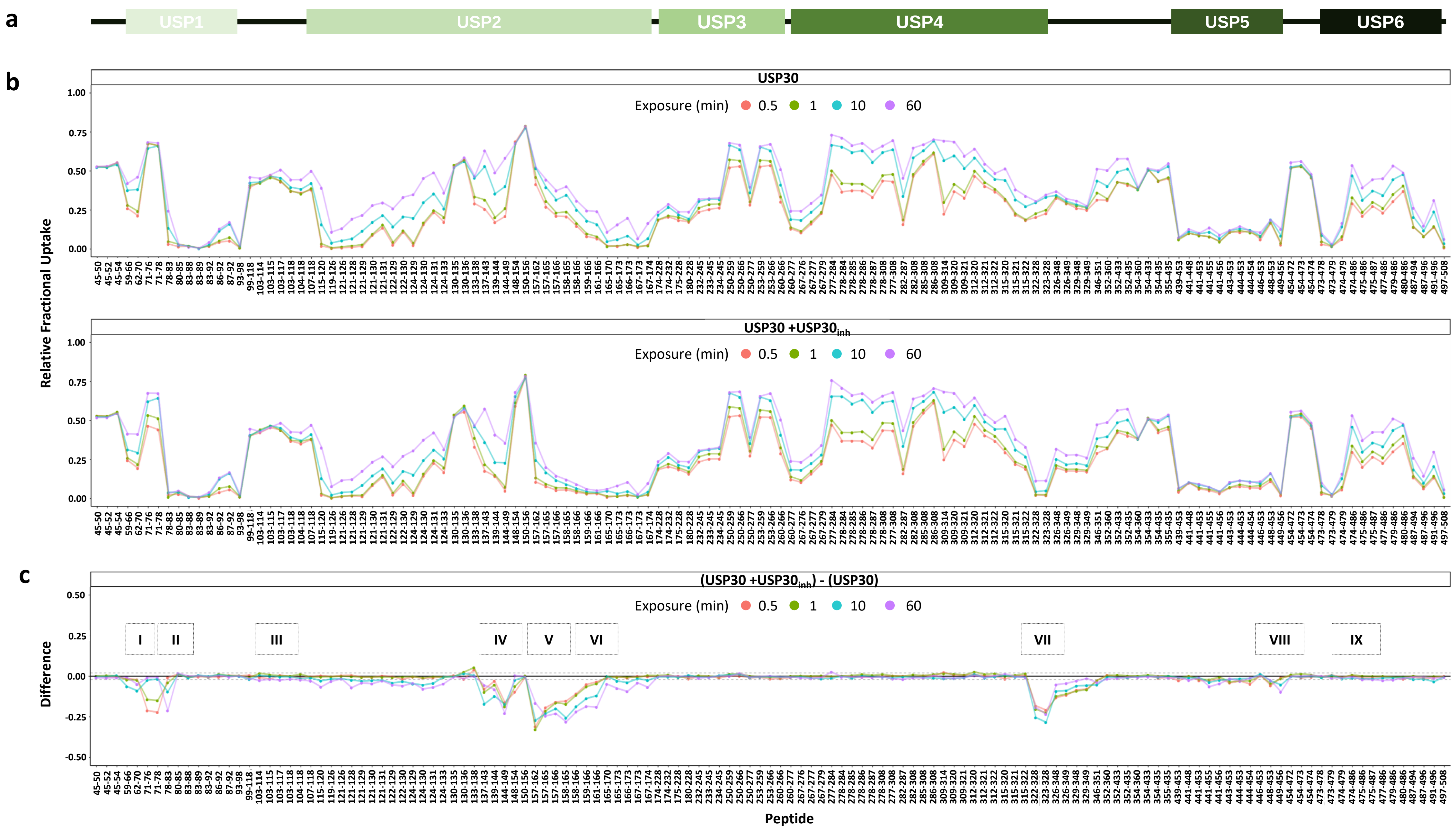

Supplemental Figure 7

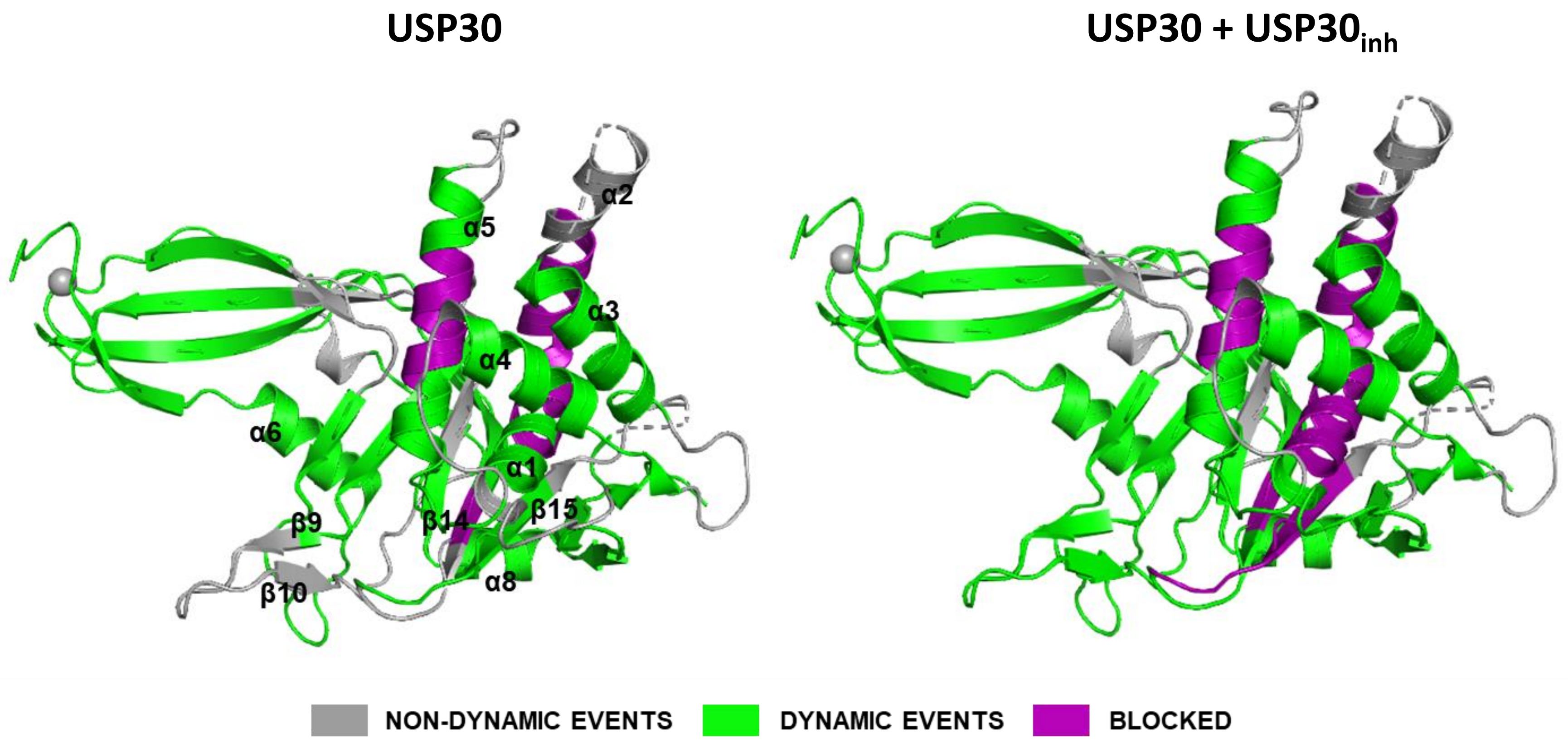

Supplemental Figure 8

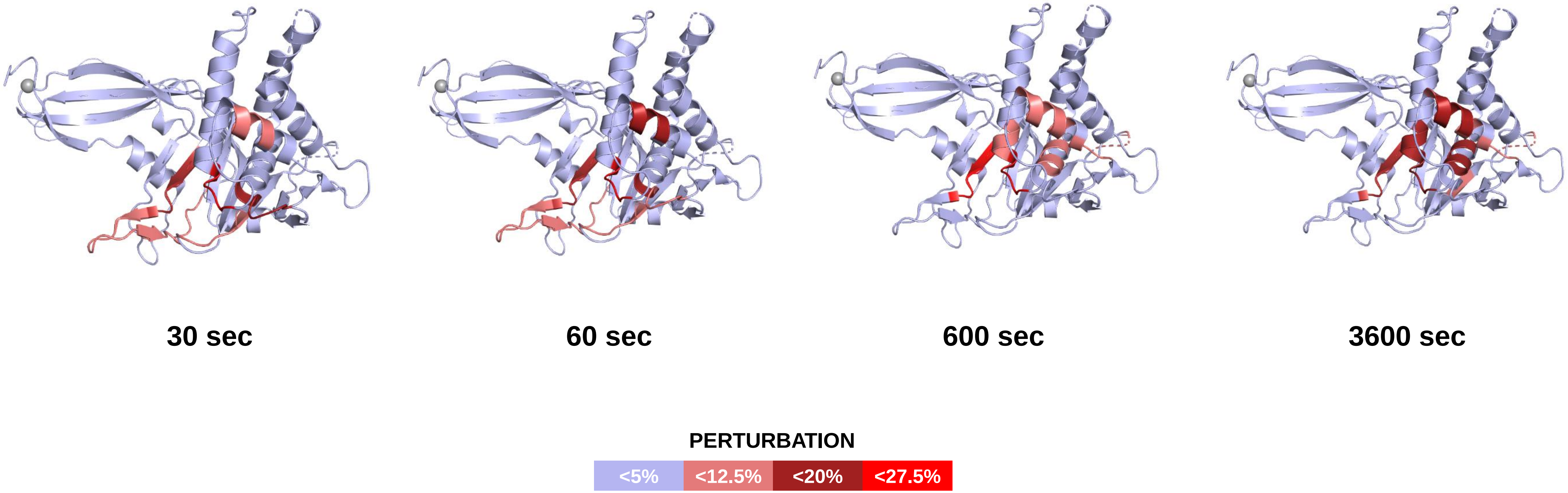

Supplemental Figure 9

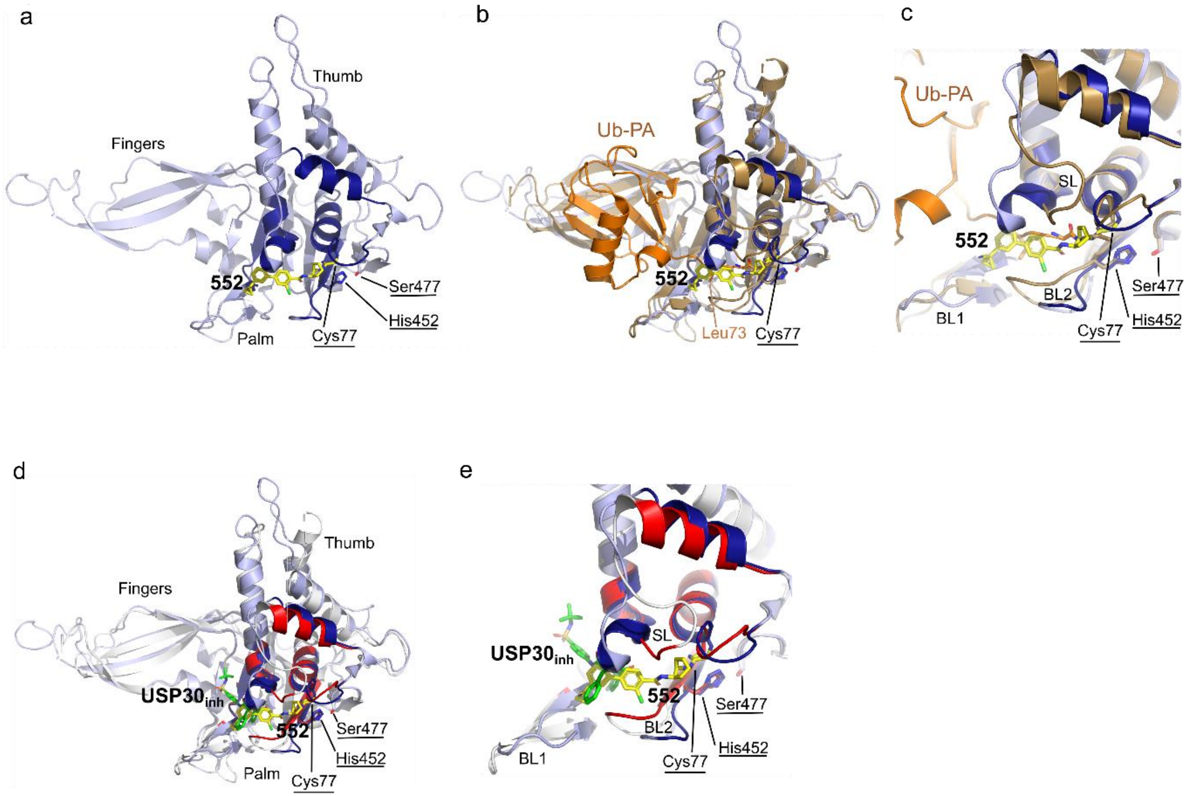

Supplemental Figure 10

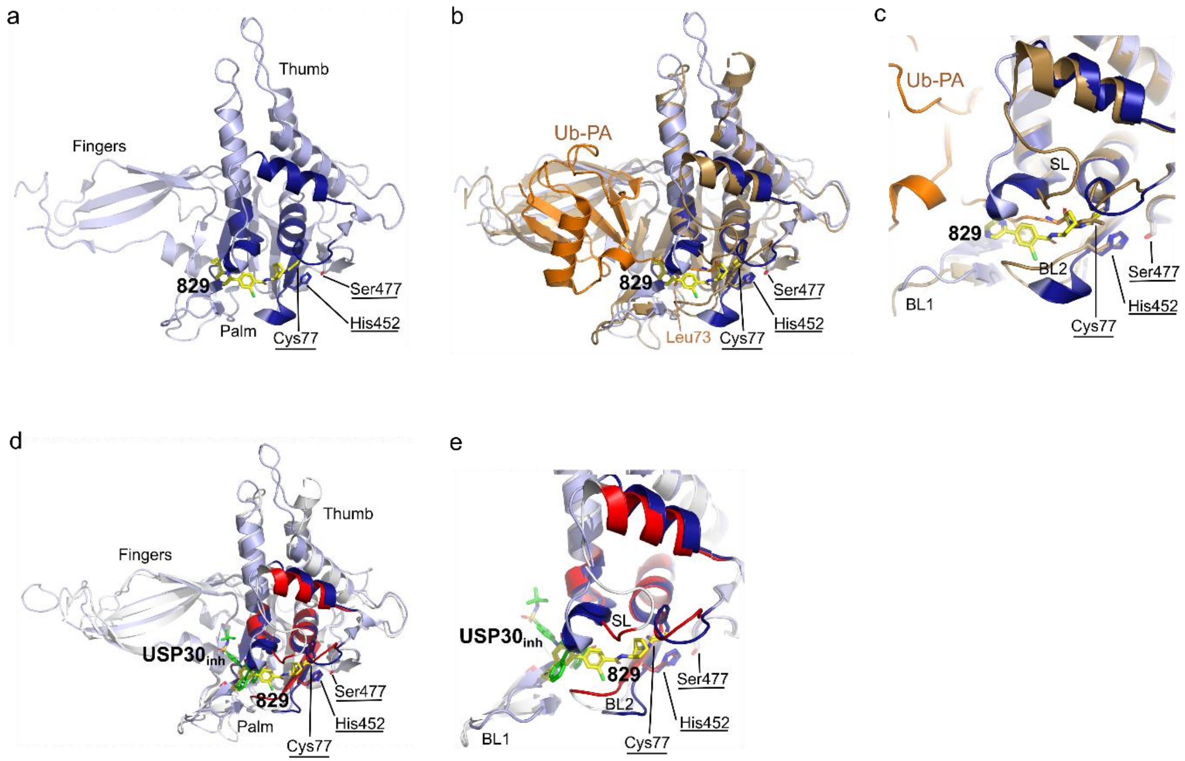

Supplement: Supplemental Figures S1–S10 [file mmc1.pdf]
