# Supplementary material for: Structural Premise of Selective Deubiquitinase USP30 Inhibition by Small-Molecule Benzosulfonamides
Source: Mol Cell Proteomics. 2023 Jun 28;22(8):100609. doi: 10.1016/j.mcpro.2023.100609 (PMC10400906; doi:10.1016/j.mcpro.2023.100609)
Supplement: Supplemental Information S1 [file mmc2.pdf]

Structural Premise of Selective Deubiquitinase USP30 Inhibition by Small-Molecule Benzosulfonamides

Darragh P O'Brien, Hannah BL Jones, Franziska Guenther, Emma J Murphy, Katherine S England, Iolanda Vendrell, Malcolm Anderson, Paul Brennan, John B Davis, Adán Pinto-Fernández, Andrew P Turnbull, and Benedikt M Kessler

SUPPLEMENTAL INFORMATION

Kinetic assays – steps to determine slow binding inhibitor constants

In case of a covalent inhibitor, IC_{50} values can be plotted against incubation time and the resulting curve can be fitted to **Eq.1** to obtain the inhibition constant K_i, and the rate of enzyme inactivation k_{inact}.

$$IC_{50}(t) = K_i\left(1 + \frac{[s]}{K_m}\right) \cdot \left(\frac{2 - 2e^{-\eta IC_{50} \cdot k_{inact} \cdot t}}{\eta IC_{50} \cdot k_{inact} \cdot t} - 1\right)$$

$$\text{Where: } \eta IC_{50} = \frac{IC_{50}\,(t)}{K_i\left(1 + \frac{[S]}{K_m}\right) + IC_{50}(t)}$$

Eq.1

In order to determine the binding kinetics of a non-covalent slow-tight binding compound progress curves were fitted to Eq.2, where [P] is product formed, t is time, v_s is the steady-state rate of the reaction, v_i is the initial rate of the reaction and k_{obs} is the rate at which the reaction converts from v_i to v_s.

$$[P] = v_s t + \frac{v_i - v_s}{k_{obs}}[1 - \exp(-k_{obs} t)]$$

Eq.2

The resulting k_{obs} values were plotted against concentration and fitted to Eq.3a to obtain k_5, k_6 and K_i^{app} .

$$k_{obs} = k_6 + \frac{k_5[I]}{K_i^{app} + [I]}$$

Eq.3a

Where k_{obs} is the rate at which the reaction converts from v_i to v_s, I is concentration of inhibitor, k_5 is the forward rate of E-I converting to E-I*, k_6 is the reverse rate of E-I converting to E-I* and K_i^{app} is the apparent equilibrium constant for the formation of E-I.

Next, an equation for converting K_i^{app} to K_i is used, assuming competitive inhibition (Eq.3b).

$$K_i = \frac{K_I^{app}}{1 + (\frac{[S]}{K_m})}$$

Eq.3b

Where K_i^{app} is the apparent equilibrium constant for the formation of EI, K_i is the equilibrium constant for the formation of E-I, S is the initial substrate concentration in the reaction and K_m is the Michaelis-Menten constant for USP30 Ub-Rho110.

The value for K_i^* was determined (Eq.3c), which is the equilibrium constant for the formation of E-I*.

$$K_i^* = \frac{K_i k_6}{k_5 + k_6}$$

Eq.3c

K_i is the equilibrium constant for the formation of E-I, k_5 is the forward rate of EI converting to EI* and k_6 is the reverse rate of EI converting to E-I*.

SUPPLEMENTAL FIGURE LEGENDS:

Figure S1: Purity profile of $USP30_{inh}$. The compound was deemed >95% pure by HPLC analysis.

Figure S2: Confirmation of USP30 complex formation with $USP30_{inh}$. The *m/z* of apo-USP30 and holo-30 (1:1 ratio of protein:compound) was measured over 75 min by Rapidfire MS. No change in *m/z* measurements indicated that the complex was stable and maintained over the time course of our experiment.

Figure S3: Inhibition of USP30 cleavage of K6-linked Di-Ubiquitin by $USP30_{inh}$ as measured by RapidFire MS. (a) 2 µM K6-linked Di-Ubiquitin in the presence and absence of 2.2 nM USP30, cleavage of Di-Ubiquitin into Ubiquitin is complete after 5 min at RT. (b) Cleavage of 1 µM Di-Ubiquitin in the presence of 0.2 nM USP30 measured over 30 min at RT. The peak at 17111.8, corresponding to Di-Ubiquitin, decreases over time while the peak for Ubiquitin at 8565 increases. (c) The same experiment as in (b) in the presence of 1 µM $USP30_{inh}$, no reduction of the 17111.8 peak is observed and no peak is seen for Ubiquitin showing that $USP30_{inh}$ is able to inhibit the cleavage of K6-linked Di-Ubiquitin.

Figure S4: Peptide map of USP30. Overall, 133 peptic USP30 peptides were selected for differential HDX-MS analysis following digestion of the unlabelled protein with pepsin. This corresponded to a sequence coverage of 96.2%, with an average peptide redundancy of 4.19. Individual peptides are represented by a blue bar on the plot.

Figure S5: HDX-MS uptake curves for individual USP30 peptides. Uptake curves for apo-USP30 are colored blue, while those of holo-USP30 are colored red.

Figure S6: Differential HDX-MS of USP30 and $USP30_{inh}$. (a) Domain organisation of USP30. (b) Relative fractional uptake of apo-USP30 (top) and holo-USP30 (bottom) by HDX-MS. All time points are individually coloured, with each measured USP30 peptide displayed on the x-axis. Peptides are sorted from the USP30 N-terminus to the USP30 C-terminus. Deuterium incorporation is mapped to the y-axis. (c) Differential deuterium uptake between holo- and apo-USP30. Regions of greatest perturbation are labeled I-IX.

Figure S7: Architecture of USP30 inferred by HDX-MS. The HDX-MS data was mapped to the crystal structure (5OHK) of both apo- and holo-USP30. Dynamic HDX behavior indicative of structural elements are colored green, while their disordered counterparts are colored grey. Regions are blocked and completely solvent inaccessible are coloured purple.

Figure S8: Temporal exchange of USP30. The HDX-MS data was mapped to USP30 at each time point and the magnitude of perturbation induced by $USP30_{inh}$ binding to USP30 color-coded. The majority of the protein is unaffected by the protein, but several regions undergo substantial (>27.5%) solvent shielding in the presence of the compound.

Figure S9. Structure of human USP30 in complex with the covalent inhibitor, 552. (a) Structure of human USP30 catalytic domain in complex with the covalent inhibitor, **552** (PDB code= 8D1T; Patent/WO/2020036940/A1), shown as a stick representation with carbon atoms colored yellow. The thumb, palm and fingers subdomains of the catalytic domain and catalytic triad (Cys77, Ser477 and His42; underlined) are highlighted. USP30 is colored light blue with regions identified in the HDX-MS analysis of USP30 in the presence of $USP30_{inh}$ mapped onto the structure and colored dark blue. (b) Superposition of USP30 in complex with Ub-PA (Ub-PA in orange, USP30 in brown; PDB code= 5OHK) on the structure of USP30 in complex with **552**. **552** is bound covalently to Cys77 via its cyanamide warhead and sterically clashes with the C-terminal tail of the Ub substrate thereby preventing Ub binding and isopeptide bond cleavage. (c) Close-up view of (b) highlighting the conformational

differences between the Ub-PA-bound and **552**-bound states including reorientation of blocking loops 1 and 2 (BL1 and BL2) and a switching loop region (SL; residues 150-162). (d) Superposition of USP30 in complex with **552** (yellow carbon atoms; USP30 is colored light blue with regions identified in the HDX-MS analysis of USP30 with $USP30_{inh}$ in dark blue) onto the docked structure of USP30 in complex with $USP30_{inh}$ (green carbon atoms; USP30 is colored grey with regions identified in the HDX-MS analysis of USP30 with $USP30_{inh}$ in red). $USP30_{inh}$ partially overlaps with **552** and putatively binds to an equivalent site in the thumb-palm cleft compared with **552**. (e) Close-up view of (d) highlighting the conformational differences between the **552**-bound and $USP30_{inh}$-modeled states including blocking loops 1 and 2 (BL1 and BL2) and the switching loop (SL) region. The benzyl moiety of $USP30_{inh}$ overlays on the cyclopropyl nitrile moiety of **552**. Conformational changes in the regions flanking the compound binding site, not accounted for in the modelling, may facilitate $USP30_{inh}$ to bind closer to the catalytic cysteine, Cys77. Figure prepared using PyMOL (The PyMOL Molecular Graphics System, Version 2.4.1, Schrödinger, LLC.).

Figure S10. Structure of human USP30 in complex with the covalent inhibitor, 829. (a) Structure of human USP30 catalytic domain in complex with the covalent inhibitor, **829** (PDB code= 8D0A; Patent/WO/2020036940/A1), shown as a stick representation with carbon atoms colored yellow. The thumb, palm and fingers subdomains of the catalytic domain and catalytic triad (Cys77, Ser477 and His42; underlined) are highlighted. USP30 is colored light blue with regions identified in the HDX-MS analysis of USP30 in the presence of $USP30_{inh}$ mapped onto the structure and colored dark blue. (b) Superposition of USP30 in complex with Ub-PA (Ub-PA in orange, USP30 in brown; PDB code= 5OHK) on the structure of USP30 in complex with **829**. **829** is bound covalently to Cys77 via its cyanamide warhead and sterically clashes with the C-terminal tail of the Ub substrate thereby preventing Ub binding and isopeptide bond cleavage. (c) Close-up view of (b) highlighting the conformational differences between the Ub-PA-bound and **552**-bound states including reorientation of blocking loops 1 and 2 (BL1 and BL2) and a switching loop region (SL; residues 150-162). (d) Superposition of USP30 in complex with **829** (yellow carbon atoms; USP30 is colored light blue with regions identified in the HDX-MS analysis of USP30 with $USP30_{inh}$ in dark blue) onto the docked structure of USP30 in complex with $USP30_{inh}$ (green carbon atoms; USP30 is colored grey with regions identified in the HDX-MS analysis of USP30 with $USP30_{inh}$ in red). $USP30_{inh}$ partially overlaps with **829** and putatively binds to an equivalent site in the thumb-palm cleft compared with **829**. (e) Close-up view of (d) highlighting the conformational differences between the **829**-bound and $USP30_{inh}$-modeled states including blocking loops 1 and 2 (BL1 and BL2) and the switching loop (SL) region. The benzyl moiety of $USP30_{inh}$ overlays on the cyclopropyl pyrazine moiety of **829**. Conformational changes in the regions flanking the compound binding site, not accounted for in the modelling, may facilitate $USP30_{inh}$ to bind closer to the catalytic cysteine, Cys77. Figure prepared using PyMOL (The PyMOL Molecular Graphics System, Version 2.4.1, Schrödinger, LLC.).

SUPPLEMENTAL TABLE LEGENDS:

Table S1: List of DIA windows. A total of 40 *m/z* windows were included in our MS acquisition method. The table lists the isolation window (*m/z*) of each of these, and their normalized AGC Target values (%).

Table S2: Protein groups identified in DIA-NN by ABPP-MS. Tab 1 lists all unique protein groups/identifications which were identified using the DIA-NN search tool for DIA datasets. In addition

to the Accession Number, Protein Name, and Gene Name, the LFQ intensity value for each protein as measured by DIA is listed for each duplicated measurement. $USP30_{inh}$ concentrations were varied from 0-25 µM concentrations in the presence of the HA-Ub probe, and compared to control samples containing no probe or USP30 inhibitor compound. Tab 2 lists equivalent LFQ data for the endogenous DUBs detected in our dataset, but at the peptide-level. Additional information here includes peptide proteotypicity, both the stripped and modified peptide sequence, as well as the peptide precursor charge state and sequence.
